# Stepped-care models for cancer symptom management: a systematic review of efficacy and cost-effectiveness

**DOI:** 10.1093/jnci/djaf153

**Published:** 2025-06-25

**Authors:** Tasnim Abdalla, Gursharan K Singh, Shiva Pourali Roudbaneh, Dorcas Serwaa, Michelle Peate

**Affiliations:** Faculty of Health and Medical Sciences, The University of Western Australia, Perth, WA, Australia; Centre for Healthcare Transformation, Faculty of Health, Queensland University of Technology, Queensland, Australia; Cancer and Palliative Care Outcomes Centre, School of Nursing, Queensland University of Technology, Queensland, Australia; Centre for Adolescent Health—Murdoch Children’s Research Institute—Royal Children Hospital, The University of Melbourne, Melbourne, Australia; Department of Obstetrics, Gynaecology, and Newborn Health, Royal Women Hospital, The University of Melbourne, Melbourne, Australia; Department of Obstetrics, Gynaecology, and Newborn Health, Royal Women Hospital, The University of Melbourne, Melbourne, Australia; Department of Obstetrics, Gynaecology, and Newborn Health, Royal Women Hospital, The University of Melbourne, Melbourne, Australia

## Abstract

**Background:**

The delivery of clinical care services using personalized health approaches is an integral component of cancer care. This review synthesized evidence on the efficacy and cost-effectiveness of stepped-care interventions delivered to manage therapy-related symptoms in cancer populations compared with care as usual (CAU).

**Methods:**

Systematic searches were conducted in MEDLINE, PsycINFO, Embase, Web of Science, Cochrane Library, National Health Service Economic Evaluation Database, and EconLit to identify studies published from January 2010 to November 2024. Peer-reviewed studies that reported outcomes of stepped interventions and CAU were included, and quality appraisal was performed using the Cochrane Risk of Bias 2 and the Risk of Bias in Non-Randomised Studies—of Interventions tools.

**Results:**

The review summarizes a total of 22 studies, involving 4588 unique adult cancer survivors. Fourteen studies identified statistically significant improvements in symptom severity and clinical outcomes comparable to those of CAU. The stepped-care group showed reduced mean severity scores for distress, insomnia, and fatigue, as well as improved stress reactions and emotional reactivity, and fewer palliative care visits. The low uptake of the intervention and inadequate assessment of comorbid symptoms have hindered the ability to draw conclusive recommendations across several studies. Four studies evaulated the cost-effectiveness of stepped-care interventions compared to CAU. Two of these studies reported significant cost savings of approximately €19 991 for each point improvement on the distress scale and lower incremental costs of approximately €3950 associated with stepped-care interventions.

**Conclusions:**

This review highlights the potential clinical and economic benefits of implementing stepped-care interventions to reduce the severity of cancer-related symptoms. Further research is warranted to assess the long-term effectiveness, cost-effectiveness, and implementation feasibility of stepped-care interventions in real-world clinical care settings serving cancer populations with diverse needs.

## Introduction

Over the past decades, the management of cancer-related symptoms has gained increasing public health attention due to the adverse psychological, physical, and financial effects of unmanaged symptoms on cancer patients and survivors.^1-3^ The presence of a single unmanaged symptom,^2^ or multiple unmanaged symptoms,^4^ can diminish the individual’s quality of life (QoL), affecting psychological and physical functioning^1^^,^^4^ and contributing to financial distress.^1^ The adverse effects of cancer can potentially extend for many years after treatment completion.^3^ Studies have identified a range of symptoms commonly experienced by cancer patients and survivors, including pain, tiredness/fatigue, sleep deprivation, loss of appetite, irritability and emotional distress, depression, poor memory, fear of recurrence, and distractibility.^5^^,^^6^ Nonclinical factors, such as limited vocational activity and social isolation, can further exacerbate and prolong these symptoms.^5^ Variations in the cancer type,^7^ stage at cancer diagnosis^8^ or therapy intensity^2^ can all affect the severity of cancer-related symptoms. Nevertheless, an elevated burden of symptoms has been observed among survivors of common cancer types (such as breast, gynaecological, and prostate cancer).^9^ Thus, effective symptom management is warranted to address the negative health and financial effects associated with the rising global burden of cancer.^10^^,^^11^

There is international recognition of the challenges associated with managing cancer (eg, high demand for clinical-care services and specialist shortages) and the need to develop and optimize cost-effective approaches to manage the short- and long-term adverse effects of cancer, including acute and persistent symptoms.^12-15^ The stepped-care approach has been endorsed in several clinical practice guidelines^16-18^ as an effective, personalized treatment approach for managing symptoms. It provides the opportunity to deliver optimal patient care that maximizes the use of scarce health-care resources (eg, specialized expertise).^19^ The stepped-care model consists of a hierarchy of interventions at varying intensity levels, matched to individual needs based on factors such as symptom severity, clinical response to initial treatment, risk of self- and other-directed aggression or history of treatment failure.^20^^,^^21^ The health system’s capacity to address individual needs depends on the availability of local resources, service structure, and patient preferences.^20^^,^^21^ This model can be implemented through a progressive or stratified approach.^17^ In the progressive approach, all patients start with the lowest intensity intervention and are stepped up to higher intensity interventions if clinically meaningful improvements are not observed.^22^ In the stratified approach, patients’ are assessed before assignment into an intervention, and subsequently matched to the appropriate intervention based on their assessment outcome.^22^ Low-intensity interventions usually involve self-paced online educational materials,^19^ while high-intensity interventions usually involve face-to-face clinician-administered therapy with or without pharmacotherapy.^23-25^

In oncology care, stepped-care models have been recommended for optimal management of depression, anxiety, and fear of cancer recurrence in adult cancer survivors, as supported by the American Society of Clinical Oncology^26^ and recent clinical pathway guidelines developed through expert consensus.^27^ Research on the effectiveness and cost-effectiveness of stepped-care interventions within cancer treatment centers and long-term follow-up clinics is an evolving area of study.^28^ Assessing the capacity of implemented interventions to optimize resource utilization and improve symptom management among cancer patients and survivors is warranted.^29^^,^^30^ This review aimed to summarize the evidence on the efficacy and cost-effectiveness of stepped-care interventions utilized to manage symptoms experienced by cancer patients and survivors.

## Methods

### Research design

The study protocol was registered in the International Prospective Register for Systematic Reviews (CRD 42022298245)^31^ and conducted in accordance with the ethical principles of the Declaration of Helsinki.^32^ The review was prepared in accordance with the Preferred Reporting Items for Systematic Reviews and Meta-Analyses guidelines.^33^

### Eligibility criteria

The selection criteria were based on the population, intervention, and outcome elements of the review objective. The target population comprised individuals who had been recently diagnosed with cancer, those currently undergoing or scheduled to undergo cancer treatment, or those who had completed their course of treatment. The primary intervention was defined as a staged or sequential therapeutic program. The comparator intervention was not specified in the selection criteria to broaden the scope of relevant evidence. The assessed outcomes were defined as changes in symptom severity, health-care costs, or frequencies of health service utilization. Unfiltered evidence from case–control studies, nonexperimental retrospective and prospective cohort studies, or randomized controlled trials was eligible for inclusion in the review. The exclusion criteria included study data not published in English, studies not available in full text, and other publication types such as case reports and conference abstracts. In addition, gray literature sources were not reviewed.

### Search strategy

A systematic literature search was conducted using the databases Medline, PsycINFO, Embase, Web of Science, Cochrane Central Register of Controlled Trials (CENTRAL), National Health Service Economic Evaluation Database, and EconLit to retrieve peer-reviewed studies published between January 2010 and October 2022. The systematic search was updated twice: first to review studies published between January 2022 and December 2023, and subsequently to review studies published from January 2024 to November 2024. The updated searches were performed in Medline, APA PsycINFO, and CENTRAL to identify additional peer-reviewed studies.

The stepped-care model emerged in the early 2000s; however, its implementation and assessment in clinical care settings remain limited.^28^ Thus, a 10-year timeline (ie, from 2000 to 2010) was considered sufficient to allow for the development of a potentially more refined model. A preliminary search for potentially relevant evidence on the health and economic benefits of stepped-care interventions implemented in cancer care settings indicated that excluding the comparison and the outcomes of the intervention from the search strategy would not significantly affect the sensitivity of the search.^34^ The following search terms were used: (neoplasm* or cancer*) AND (“stepped care” or stepped-care or “adaptive care” or “sequential care” or stage-based or “stratified care”). Database-specific medical subject headings and Boolean operators were used to optimize the retrieval of relevant articles.^35^^,^^36^ The reference lists of eligible studies were manually searched to identify other relevant studies. A comprehensive summary of the search strategy can be found in Table S1.

### Study selection

The screening process was managed using the reference management systems EndNote X21 (Clarivate, United States) and Covidence (Veritas Health Innovation, Australia). Four reviewers (T.A., G.K.S., S.P.R., D.S.) independently screened the titles and abstracts of nonduplicate articles to assess their relevance. Discrepancies in eligibility decisions were resolved through discussion with a senior reviewer (M.P.). Four reviewers (T.A., G.K.S., S.P.R., D.S.) conducted the full-text review of all eligible and potentially eligible studies, with each article independently assessed by 2 reviewers. Interrater agreement between reviewers was assessed using the unweighted Cohen’s Kappa statistic (*κ*), which estimates the level of agreement beyond chance.^37^ Kappa statistic ratings are defined as follows: substantial (*κ* > 0.60), moderate (*κ* 0.41-0.60), poor to fair (*κ* < 0.40).^37^ In this study, the interrater statistic indicated substantial agreement between reviewers’ decisions (*κ* statistic = 0.7).

### Data extraction and synthesis

Details of the study design were extracted, including participant characteristics, geographical location, recruitment setting, symptoms assessed, assessment scales used, perspective of economic evaluation, estimated costs, research design, and duration of outcome assessment. Details of the administered interventions were also extracted, including the number and description of stepped-care components, mode of delivery, clinical step-up criteria, description of usual care or control interventions, and main findings. One reviewer (T.A.) extracted the data using a structured Excel extraction form. Subsequently, 3 reviewers (G.K.S., S.P.R., and D.S.) evaluated the accuracy and completeness of the extracted data. A narrative synthesis was performed to summarize evidence on the effectiveness and cost-effectiveness of the stepped-care models relative to care as usual (CAU), where available. The following information was synthesized: changes in symptom severity scores, proportions of patients with improvements in assessed symptoms or quality-of-life domains, frequency of health-care service utilization, and health and nonhealth cost savings and cost-effectiveness. Descriptive statistics (means, *P*-values, and SD) of the study characteristics and effect measures were also reported.

### Risk of bias (quality) assessment

The Cochrane Risk of Bias (version 2, RoB2) tool was used to evaluate the strength of evidence in studies with a randomized controlled design.^38^ Risk of Bias 2 assesses the risk of bias using signaling questions within 5 domains: (1) bias arising from the randomization process, (2) bias due to deviations from intended interventions, (3) missing outcome data, (4) bias in the measurement of the outcome, and (5) bias in the selection of the reported result. The judgment for each domain is compiled into an overall judgement, classified as “low risk of bias,”, “some concerns,” or “high risk of bias,” depending on the *worst* judgment in any of the domains.^38^ The Risk of Bias In Non-randomised Studies—of Interventions (ROBINS-I) tool was used to evaluate the strength of evidence in studies with a non-randomized design.^39^ Risk of Bias In Non-randomised Studies—of Interventions assesses internal validity through 7 signaling domains related to the experiment stages: preintervention (addressing bias due to uncontrolled confounding and selection of participants into the study), intervention (addressing classification of interventions), and postintervention (addressing deviations from intended interventions, handling of missing data, measurement of outcomes, and selection of the reported result).^39^ Four reviewers (T.A., G.K.S., S.P.R., and D.S.) independently assessed the quality of the evidence. A senior reviewer (M.P.) was available to discuss unresolved discrepancies in decisions and facilitate consensus. Although no strict inclusion criteria were used based on the quality assessment, recommendations were formulated based on the reliability of the reviewed evidence.

## Results

### Literature search and study characteristics

**Figure 1.**
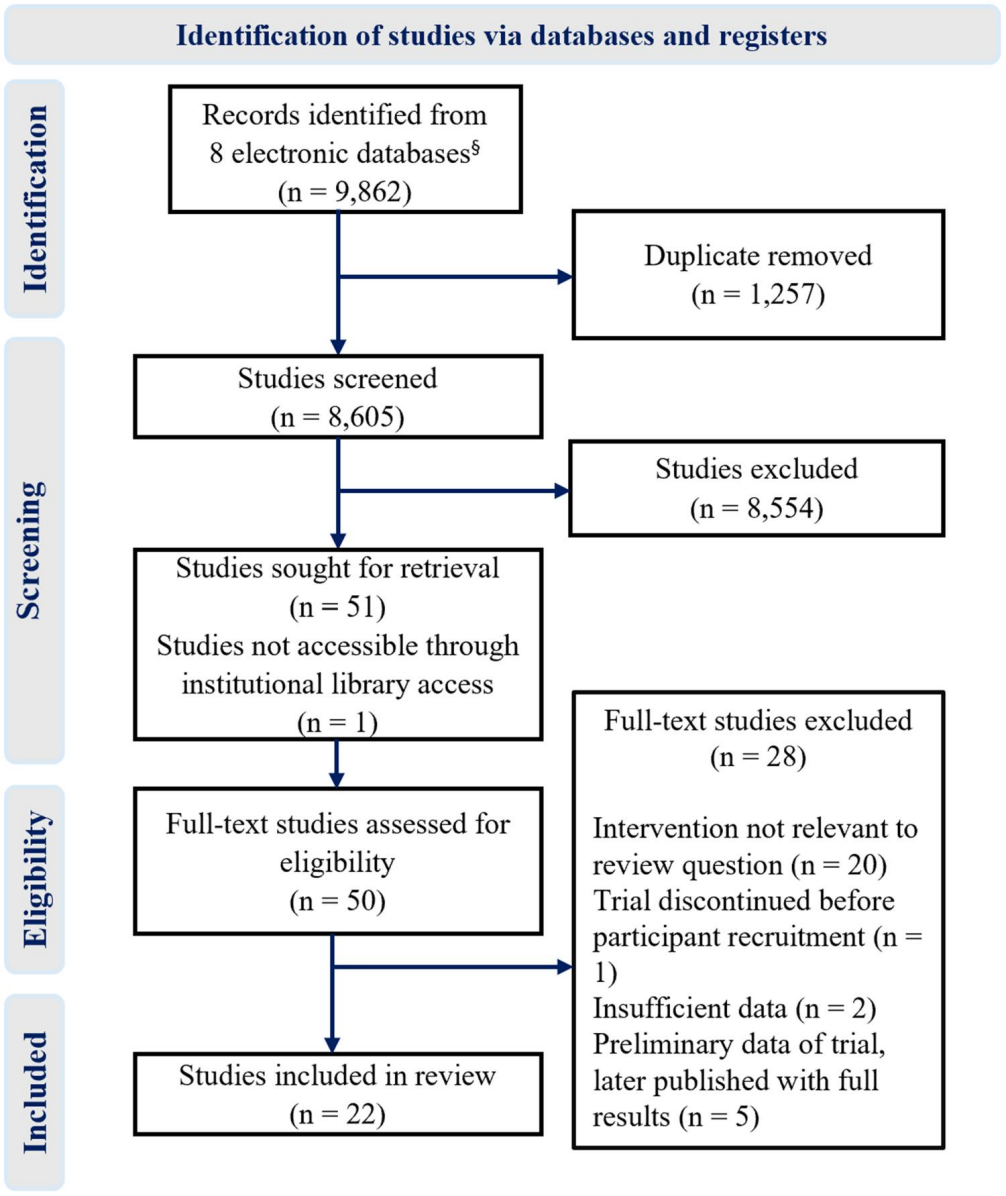
Preferred reporting items for systematic reviews and meta-analyses diagram of included studies. ^§^Updated searches from October 2022 to December 2023 were performed in 3 of the 8 databases.

The systematic search identified 9862 published abstracts; 22 peer-reviewed studies met the inclusion criteria.^40-61^  Figure 1 summarizes the selection and exclusion of the identified articles at different stages of the review. Prospective randomized controlled trial (RCT) was the predominant design in 17 studies. Five studies evaluated the intervention using nonrandomized study designs, including 3 single-group sequential designs,^43^^,^^56^^,^^57^ 1 prospective nonrandomized design,^49^ and 1 retrospective cost-effectiveness analysis.^59^

Sixteen studies analyzed the effect of the intervention in unique participant samples (Table 1),^40-43^^,^^45^^,^^48-50^^,^^52-57^^,^^60^^,^^61^ while 6 studies evaluated the outcomes of the intervention by examining the participants of an existing RCT.^44^^,^^46^^,^^47^^,^^51^^,^^58^^,^^59^ Of the 16 distinct RCTs, 4 summarized outcomes in cancer cases irrespective of their treatment status.^43^^,^^55^^,^^56^^,^^60^ Ten of the 16 studies examined participants diagnosed with any cancer type.^40^^,^^43^^,^^49^^,^^50^^,^^53-57^^,^^60^ Six (out of 16) studies examined participants with specific cancer types (primarily colorectal, lung, head, and neck tumors).^41^^,^^42^^,^^45^^,^^48^^,^^52^^,^^61^ Four (out of 16) studies exclusively examined cases of advanced and metastatic cancers.^49^^,^^52^^,^^54^^,^^61^ Stratified outcomes by other cancer-related characteristics were rarely reported. One study, however, briefly investigated whether referral to consultation-liaison services and improvements in emotional well-being differed by the stage of cancer diagnosis, comparing patients with early-stage (I/II) vs advanced-stage (III/IV) disease.^53^

**Table 1.** Characteristics of randomized controlled trials and non-randomized experimental studies included in the systematic review.

Study (year) location	Outcome assessment		Participants’ characteristics		Study design		
	Symptoms	Measurement scale/s	Study population	Mean age (in years)	Study type (analysis sample size)	Recruitment setting	Assessment duration (in months)
Braamse et al. (2016) Amsterdam and Zwolle, the Netherlands	Psychological distress; Emotional functioning	Hospital Anxiety and Depression (HADS)—total, anxiety (HADS-A) and depression (HADS-D); and other measures^a^	Hematological cancer patients scheduled for treatment with high-dose chemotherapy and autologous stem cell transplantation; Excluded cases with <3 months life expectancy and insufficient Dutch language skills	54.4	RCT (SC=47, CAU-48)	Two hematology departments	10
Krebber et al. (2016) Amsterdam, the Netherlands	Untreated psychological distress; Health-related quality of life (HRQoL)	HADS—total and subscales (HADS-A and HADS-D); other measures^b^	Head, neck, or lung cancer cases treated with curative intent at least 1 month prior, and with indicators of mild to severe symptoms of distress; Excluded cases with cognitive impairment, lack of motivation for treatment, high suicide risk, psychotic and/or manic symptoms, current or recent treatment for distress or a psychiatric disorder, or insufficient knowledge of Dutch language	62.0	RCT (SC=75, CAU=81)	Outpatient clinics	16 approx.
Steel et al. (2016) Pittsburgh, Pennsylvania, United States	Fatigue; Depression; Chronic pain; HRQoL	The Functional Assessment of Cancer Therapy-Fatigue (FACT-Fatigue); Centre for Epidemiological Studies Depression Scale (CES-D); Brief Pain Inventory (BPI); FACT-General	Advanced or metastatic cases of hepatocellular, cholangiocarcinoma, gallbladder, neuroendocrine, and pancreatic carcinoma, or other primary cancers with liver metastases, and with life expectancy >1 year; Excluded cases with cognitive dysfunction, suicidal ideation, or insufficient command of English	61.0	RCT (SC=124, CAU=100)	Oncology outpatient clinic and/or hospital	6
Singer et al. (2017) Leipzig, East Germany	Psychological distress	HADS; Other measures^c^	Any cancer patient admitted to hospital wards offering standard consultation, followed by psycho-oncological care as needed, and with sufficient proficiency in the German language	63.7	RCT (SC=570, CAU=442)	13 inpatient wards, university medical center	6
Turner et al. (2017) Australia	Psychological distress; Pain/discomfort; Unmet sexual needs	HADS; other measures^d^	Any cancer patient (irrespective of disease and treatment status); Excluded cases with severe depression, at risk of depression or currently taking depression medication, cognitive impairment, <6 months life expectancy, insufficient command of English, or significant medical complexities	58.7	RCT (SC=177, CAU=181)	Four cancer treatment centers	2.5
Arving et al. (2019) Bergen, Norway	Stress reactions; Psychological distress; Emotional reactivity	The Impact of Event Scale (IES) for avoidance behaviour (IES-A) and intrusive thoughts (IES-I); HADS; Everyday Life Stress Scale (ELSS)	Newly diagnosed cancer patients, with no previous history of a cancer diagnosis or an ongoing psychiatric condition	61.0	RCT (SC=145, CAU=146)	Oncology department	6.5
Jansen et al. (2019) Amsterdam, the Netherlands	Psychological distress; Emotional functioning	HADS—total and subscales (HADS-A and HADS-D); other measures^b^	Head, neck, or lung cancer cases who underwent cancer treatment at least 1 month prior, and had increased levels of distress, anxiety, or depression symptoms	62.3	RCT (SC=75, CAU=81)	A university medical center	16 approx.
Schuurhuizen et al. (2019) the Netherlands	Psychological distress; HRQoL	HADS; Distress Thermometer/Problem List (DT/PT); EORTC QLQ-C30	Cases with metastatic colorectal cancer who were scheduled for chemotherapy treatment, had >3 months life expectancy, and no severe psychopathology or recent psychotherapy	66.1	RCT (SC=184, CAU=165)	Medical oncology departments of 16 hospitals	12
Hauffman et al. (2020) Mid-Sweden	Psychological distress; Insomnia; Fatigue; Posttraumatic stress; HRQoL	HADS; STAI-S; Asberg Depression Rating Scale (MADRS-S); the psychometric properties of the Environmental Reward Observation Scale (EROS); other measures^e^	Newly diagnosed cases of breast, colorectal, or prostate cancer, as well as relapsed colorectal cancer cases, with a self-reported HADS-A or HADS-D score >7; Excluded cases with <3 months life expectancy, those with severe depression or suicide risk, and those with cognitive or functional impairment	57.2	RCT (SC=124, CAU=121)	Four hospitals	10
Lynch et al. (2020) Melbourne, Australia	Fear of cancer recurrence; Fear of disease progression	Fear of Cancer Recurrence Inventory Short Form (FCRI-SF); Fear of Progression Questionnaire Short Form (FoP-Q-SF)	Histology confirmed stage IV metastatic melanoma cases who received at least 6 months of immunotherapy and/or targeted therapy; and had a complete, partial, or stable response to the administered therapy; Excluded cases considered too unwell by their treating team or had insufficient English proficiency	61.4	Prospective, nonrandomized (SC=25, CAU=36)	Two metropolitan hospital-based outpatient clinics	4 approx.
Zhou et al. (2020) Boston, United States	Insomnia; Mood disturbance	ISI^e^; other measures^f^	Cancer survivors with no history of active therapy (excluding chemoprevention) in the past 12 months, or a scheduled cancer therapy or surgery within the next 6 months; and with ISI score ≥12	54.4	Single-group, sequential (51)	A cancer center (and advertisements, mailed invitations and oncologist referrals)	5 approx.
Savard et al. (2021) Quebec, Canada	Insomnia; Psychological distress; Fatigue; HRQoL	ISI; other measures^g^	Any individual with a nonmetastatic cancer diagnosis who either had an ISI score ≥8 or regularly uses a psychotropic medication to aid sleep, lives within 50 km of the research center, and is proficient in French	55.2	RCT (SC=118, CAU=59)	A radio-oncology department	12
Schutte et al. (2021) Amsterdam, the Netherlands	Lack of sexual interest and enjoyment; Depression; Anxiety HRQoL	EORTC QLQ-H&N35 - sexual interest and enjoyment; other measures^h^	Head and neck cancer or lung cancer cases treated with curative intent at least 1 month earlier, and were experiencing depression or anxiety (HADS—total score >14, HADS-A or HADS-D score >7); Excluded cases with cognitive impairment, no motivation for treatment, high suicide risk, psychotic or manic symptoms, current treatment for distress, or insufficient knowledge of Dutch	61.8	RCT (SC=67, CAU=67)	Outpatient clinic at a university medical center	12
Borrayo et al. (2023) Colorado, United States	Psychological distress; Emotional distress; Coping self-efficacy; Perceived stress; HRQoL	HADS; Patient-Reported Outcomes Measurement Information System for cancer (PROMIS); Perceived Stress Scale (PSS); Coping Self-Efficacy (CSE); FACT-G	Recently diagnoses cases of head and neck, or lung cancer, who are proficient in English or Spanish, come from a low-income background, and have no or minimal insurance; Excluded cases with impaired decision making, those belonging to vulnerable groups, those not receiving treatment onsite, or those with auditory impairments	66.0 (median)	RCT (SC=120, CAU=123)	Five cancer treatment sites	6
Diggens et al. (2023) Melbourne, Australia	Insomnia	ISI; Epworth Sleepiness Scale (ESS); other measures^i^	Participants with a history of cancer diagnosis (irrespective of time since initial diagnosis, cancer stage, or presence/absence of anti-cancer treatment at enrolment), and an ISI score >7 and/or ESS score >10	55.4	Single-group, sequential (87)	Three metropolitan hospitals, and 6 outpatient oncology clinics	2.1
Igelstrom et al. (2023) Mid-Sweden	Anxiety or depression; Insomnia; Fatigue; HRQoL	HADS; STAI-S; Asberg Depression Rating Scale (MADRS-S); the psychometric properties of the Environmental Reward Observation Scale (EROS); other measures^e^	Recently diagnosed cases of breast, colorectal or prostate cancer or recurrent cases of colorectal cancer, with a self-reported HADS-A or HADS-D score >7; Excluded cases with <3 months life expectancy, severe depression, suicide risk, cognitive impairment, functional status <40, or insufficient knowledge of Swedish	57.2	RCT 18 months (SC= 62, CAU=68) 24 months (SC=55, CAU=63)	Four hospitals	24
Williams et al. (2023) Melbourne, Australia	Fatigue; HRQoL	FACIT-F; EQ-5D-5L^d^	Cases with a history of cancer or receiving long-term treatment for a stable cancer diagnosis, who reported moderate to severe fatigue; Excluded cases with an interfering medical disorder (including significant sleep disorder) or a psychological issue	64.3	Single-group, sequential (19)	A cancer center	3 approx.
Temel et al. (2024) Boston, Philadelphia, and Durham, United States	HRQoL	FACT-Lung (FACT-L)	Recently diagnosed cases of advanced lung cancer or mesothelioma within the past 12 weeks, not receiving curative therapy, with no or moderate functional impairment, and the ability to comprehend English or Spanish; Excluded cases receiving outpatient palliative care, enrolled in hospice, or with disabling cognitive or psychiatric conditions	66.5	RCT (SC=250, CAU=257)	Three medical centers	12 approx.

Cost-effectiveness of stepped-care							
Study (year) Location	Perspective and estimated costs	Economic evaluation and symptoms	Study population	Mean age (in years)	Study type (sample size)	Recruitment setting	Assessment duration (in months)
Jansen et al. (2017) Amsterdam, the Netherlands	Societal perspective; Costs of intervention, direct medical care, direct nonmedical care, and indirect nonmedical care	Mean cumulative costs; mean number of quality-adjusted life years (QALYs) measured using EuroQol-5 Dimension for quality of life	Head, neck, or lung cancer cases who received curative treatment at least 1 month prior, and had HADS—total score >14, HADS-A or HADS-D score >7; Excluded cases with cognitive impairment, lack of motivation for treatment, high suicide risk, psychotic symptoms, or those currently receiving treatment for distress	62.3	RCT (SC=75, CAU=81)	A university medical center	16 approx.
Alili et al. (2020) The Netherlands	Societal perspective; Costs of cancer treatment, medication, other health-care, informal care, productivity loss, and the intervention	Number of QALYs measured using EuroQol-5 Dimension for quality of life and societal costs in Euros (2016 CPI); Psychological distress: HADS; DT/PT	Patients diagnosed with terminal colorectal cancer, scheduled for chemotherapy treatment, with a >3 months life expectancy, and without severe psychopathology or psychotherapy in the last 3 months	66.0	RCT (SC=184, CAU=165)	Medical oncology departments at 16 hospitals	12 approx.
Pilehvari et al. (2024) Boston, United States	Payer’s perspective; Group sessions delivery costs, computed from the unit price of each resource utilized during the session	Incremental cost-effectiveness ratio, comparing the mean costs and effects of interventions delivered in trial I (a three-session CBT-I program) and trial II (stepped-care model)	Adult cancer survivors (breast cancer survivors comprised 65.2% of the 3-session CBT-I group and 63.6% of the stepped-care group), and with ISI score ≥ 12	54.1	Retrospective cost-effectiveness analysis (SC=51, three-session CBT-I group=23)	A cancer center (and advertisements, mailed invitations and oncologist referrals)	4 approx.
Steel et al. (2024) Pittsburgh, Pennsylvania, United States	Provider’s perspective (activity-based costing); Health care use	Cost difference per patient per year and cost savings; Depression assessed using CES-D, pain and fatigue assessed using a single-item 0-10 scale	Participants with a clinically or radiologically confirmed cancer, and a clinical meaningful level of depression, pain, or fatigue; Excluded cases with insufficient English comprehension, a psychiatric symptom (thought disorder, delusions, hallucinations), or suicidal ideation	65.7	RCT (SC=237, CAU=222)	29 oncology outpatient clinics	12

a European Organisation for Research and Treatment of Cancer Care Questionnaire—emotional, physical, and role functioning (EORTC QLQ-C30); The Spielberger State-Trait Anxiety Inventory (STAI).

b EORTC Head and Neck Module (EORTC QLQ-H&N35); EORTC Lung Cancer Module (EORTC QLQ-LC13); EORTC Patient Satisfaction with Care (EORTC IN-PATSAT32).

c Patient Health Questionnaire Short Form (PHQ‐9); Generalized Anxiety Screener 7 (GAD-7); EORTC QLQ-C30.

d FACT-General; the EuroQol 5 Dimension 5 Level for quality of life (EQ-5D-5L); Demoralization Scale.

e Insomnia Severity Index (ISI); EORTC QLQ-C30; FACIT-F; Post-Traumatic Stress Disorder Checklist for Civilians (PCL-C).

f Profile of Mood States-Short Form (POMS-SF).

g HADS; Fatigue Inventory Symptom (FSI); EORTC QLQ-C33.

h HADS (total, HADS-A, HADS-D); EORTC QLQ-C30.

i STOP-BANG Sleep Apnea Questionnaire; Restless Leg Screening Tool (RLST).

The review summarized the outcomes observed in 4588 unique participants (primarily adults aged 18 years), comprising diagnosed cancer patients^40-43^^,^^45^^,^^49^^,^^50^^,^^52-56^^,^^60^^,^^61^ or cancer survivors with a history of oncologic treatments.^57^^,^^59^ The participants were recruited from various health-care settings (including oncology departments, hospitals, and medical care clinics) in Australia, Canada, Denmark, Germany, the Netherlands, the United States, Norway, and Sweden. The sample sizes of the reviewed studies ranged from 19^56^ to 1012 participants.^53^ Females constituted 49.8% of the total unique participants. The mean age of participants was 63.8 years (range 54.4-66.5 years).^40^^,^^42^^,^^43^^,^^45^^,^^48-50^^,^^52-55^^,^^57^^,^^60^^,^^61^ The studies evaluated the effects of interventions over varying durations, ranging from 2.1^43^ to 24^58^ months. Several studies excluded participants with characteristics that might confound or distort the intervention’s effect, including current psychotherapy or psychotropic medications, suicidal risk, specific medical complexities (eg, severe psychopathology, cognitive impairment, sleep apnea, or any disease burden), or insufficient English-language proficiency (Table 1).

### Risk of bias within studies

**Figure 2.**
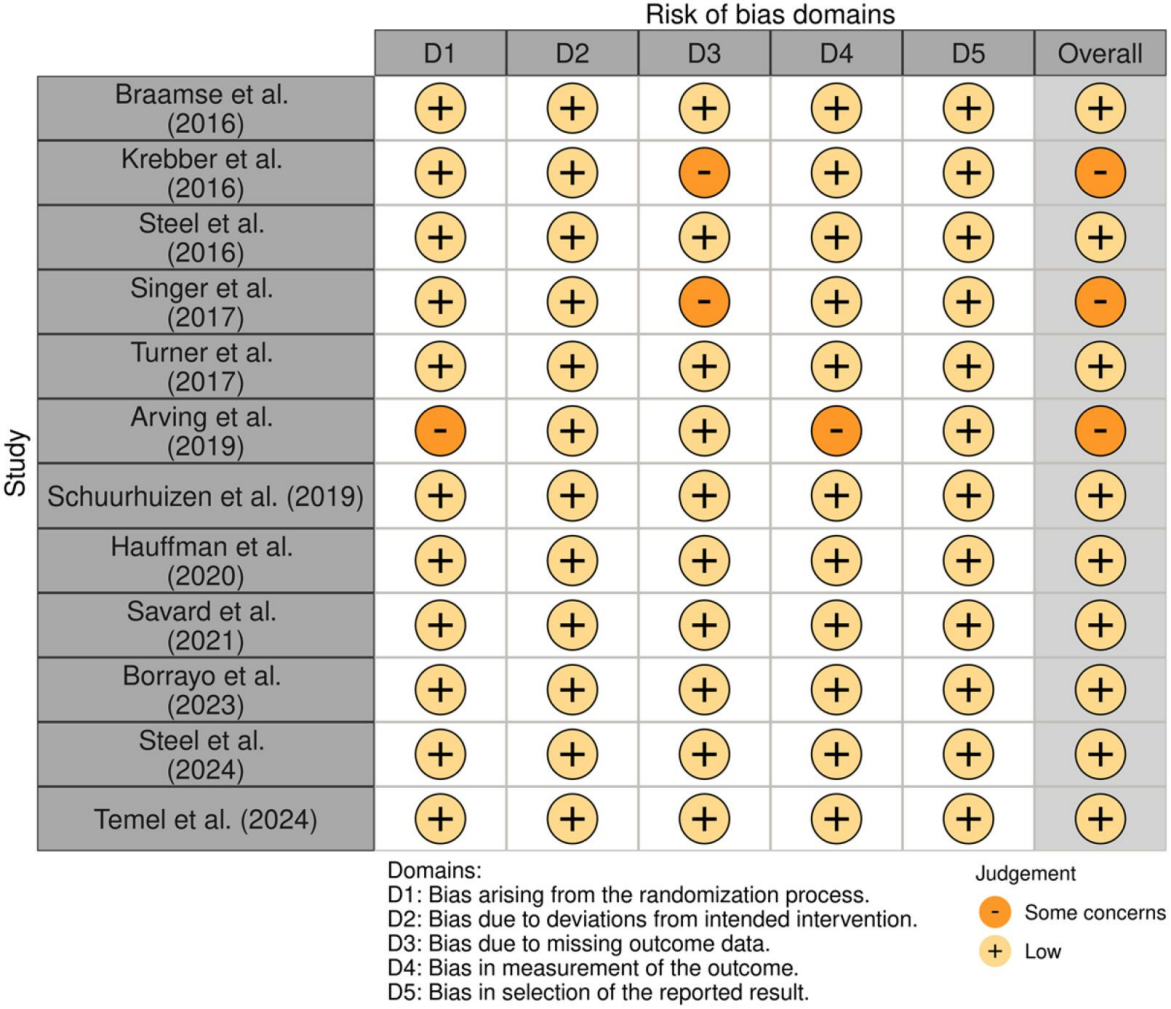
Risk of bias assessment for distinct randomized controlled trials, according to the domains of the Cochrane risk-of-bias tool (version 2).

**Figure 3.**
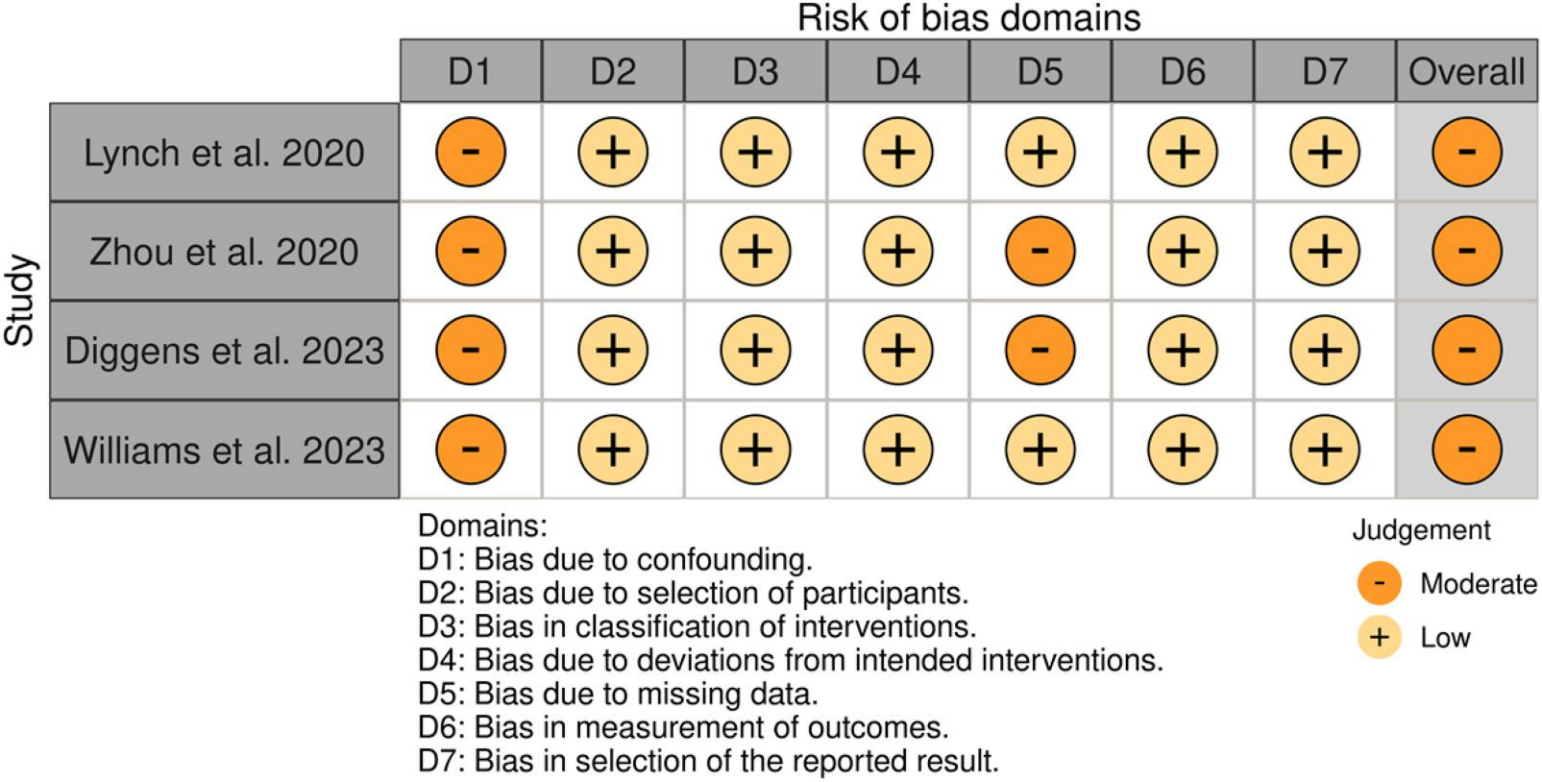
Risk of bias assessment for distinct nonrandomized experimental studies, according to the domains of the Risk of Bias in Non-Randomised Studies—of Interventions.

The risk of bias was judged to be low in 83% of the included RCTs (Figure 2). There was some concern for bias in 2 studies related to the randomization process (specifically the concealment of allocation),^40^ the percentage of missing outcome data,^48^^,^^53^ and the measurement of the outcome.^40^ The risk of bias across the single-arm studies testing the feasibility of stepped intervention was judged to be moderate, primarily due to insufficient control for key confounding factors^43^^,^^49^^,^^56^^,^^57^ and the high dropout rates during the higher intensity steps (Figure 3).^43^^,^^57^

### Assessed outcomes

Psychological distress (primarily defined as the presence of anxiety or depression) was the most commonly examined symptom,^40-42^^,^^45-48^^,^^50^^,^^52-55^^,^^60^^,^^61^ followed by insomnia,^43^^,^^45^^,^^50^^,^^57-59^ health-related quality of life (HRQoL),^41^^,^^45^^,^^50^^,^^54^^,^^61^ and fatigue^45^^,^^50^^,^^54^^,^^56^ (Table 1). The main measurement scales used to assess these symptoms were the Hospital Anxiety and Depression Scale (HADS, anxiety HADS-A, and depression HADS-D),^40-42^^,^^44-48^^,^^50^^,^^52^^,^^53^^,^^55^ the Insomnia Severity Index (ISI),^43^^,^^45^^,^^50^^,^^57^ the Functional Assessment of Cancer Therapy-Fatigue (FACT-Fatigue), and the European Organisation for Research and Treatment of Cancer Quality of Life Questionnaire (EORTC QLQ-C30).^45^^,^^48^^,^^50^^,^^51^ In one study, palliative care clinicians documented the pattern of palliative care service use among participants in the trial arms.^61^ The cost-effectiveness of stepped-care interventions was assessed in 4 studies by estimating the health and economic benefits measured in quality-adjusted life years (QALYs)^44^^,^^46^ and health-care costs^59^^,^^60^ (Table 2).

**Table 2.** Summary of stepped-care models, their outcomes, and economic evaluations.

Study *(year)* Location	Stepped-care (SC) intervention				Care as usual (CAU)	Effect of SC compared with CAU
	Number and components of SC interventions	SC approach	Delivery mode	Step-up criteria		
Braamse et al. (2016) Amsterdam and Zwolle, the Netherlands	Step 1—Watchful waiting (6 weeks) Step 2—Internet-based self-help problem-solving therapy (4 weeks) Step 3—Individual face-to-face counseling, psycho-pharmacological treatment, or referral to other services	Stratified	Mixed	Persistent psychological distress (HADS score ≥13 or Lastmeter score ≥5 score) or patient preference to receive a higher-intensity intervention	Emotional support was provided during follow-up visits on an ad hoc basis by hematologists/oncologists and nurses; reported problems were resolved by these professionals or referred to other services as needed	Inconclusive evidence of SC efficacy, due to the low severity and prevalence of psychological distress at baseline, low uptake of the intervention, and the high quality of CAU
Krebber et al. (2016) Amsterdam, the Netherlands	Step 1—Watchful waiting (2 weeks) Step 2—Guided self-management via the internet or a booklet (5 weeks) Step 3—Face-to-face problem-solving therapy (6 sessions maximum) Step 4—Specialised psychological therapy or psychotropic medications	Progressive	Mixed	Persistent symptoms (HADS-A score > 7; HADS-D score > 7); Step 4 intervention offered if the patient consented	No psychosocial care for 52 participants; Psychological treatments and/or psychotropic medications for 20 participants	Higher recovery rate posttreatment (54.8% vs 29.2%, *P* <.05), but not at the end of follow-up (recovery rate at 12 months 45.5% vs 36.5%, *P* > .05); Clinical and statistically significantly higher scores on social functioning and contact, and sexuality problems (all > 10-point difference, *P*<.05); Higher improvements among participants with a depression or anxiety disorder compared with those without such a disorder (*P*<.05)
Steel et al. (2016) Pittsburgh, Pennsylvania, United States	Step 1—Internet-based, guided self-management using psycho-educational information, telephone support (every 2 weeks), and clinic consultations (every 2 months) Step 2—Internet-based, guided cognitive-behavioral therapy (CBT) and/or recommendations for medication if referred to CBT	Progressive	Internet-based	Persistent symptoms (CES-D ≥16; BPI ≥3; or FACT-Fatigue ≥26)	Enhanced CAU: participants were screened for depression, fatigue, and pain. Symptom education and suicidality assessment were provided. Referral options were often based on their treatment preference. Participants with a CES-D score ≥16 or a pain indicator > 5 on a 0-10 scale were referred to a community-based mental health professional or their general practitioner	Large effect size for HRQoL (Cohen’s *d* =0.9), medium effect sizes for pain (Cohen’s *d* =0.6) and depression (Cohen’s *d* =0.7), and a small effect size for fatigue (Cohen’s *d* =0.2) were observed at 6 months compared with CAU; Cancer treatment exposures had no significant influence on the intervention effect across cases (χ^2^ 0.8, *P* > .05)
Singer et al. (2017) Leipzig, East Germany	Step 1—Screening for distress, pain, and fatigue Step 2—Routine clinical consultation and treatment plan discussion Step 3—Referrals to hospital services upon clinician–patient shared agreement	Progressive	In-person	Presence of severe distress (PHQ-9 or GAD-7 score ≥10); fatigue (EORTC QLQ-C30 fatigue ≥75th); pain (EORTC QLQ-C30 pain ≥90th percentile); and agreement to engage in a treatment plan	No routine screening for distress; Doctors without specific training in detecting distress or providing emotional support referred participants to specialized psychological care at their discretion	Non-significant difference in mean HADS scores at 6 months (SC=9.4 vs CAU=9.3, *P* > .05); SC enhanced referral to psychosocial services (adjusted OR 10.0, 95% CI=2.8 to 35.9, *P*<05); 35% vs 8% of participants with poor well-being at baseline referred to psychosocial services (adjusted OR 4.8, 95% CI=1.7 to 13.4, *P*<.05)
Turner et al. (2017) Australia	Step 1—Guided self-management using educational material Step 2—Intervention delivered by trained health professionals (first-line cancer care professionals) Step 3—Specialized treatment	Stratified	In-person	Persistent symptoms Step 1 (HADS score <8) Step 1 - > 2 (HADS score 8-21) Step 2 - > 3 (HADS score ≥22)	Doctors without specialized training referred participants to specialized psychological care at their clinical judgement	Non-significant difference at 2.5 months for depression, anxiety, pain/discomfort, or unmet sexual needs (*P* > .05); Participants with a baseline HADS score ≥15 and disease progression showed greater improvements (*P*<.05)
Arving et al. (2019) Bergen, Norway	Step 1—Counseling session and follow-up (including dissemination of educational materials on self-management of stress symptoms) Step 2—Stress management intervention and 3-8 additional sessions	Progressive	In-person	Persistent symptoms (HADS score ≥8 and/or IES score ≥9)	Regular consultations with family doctors and hospital staff, and participation in a rehabilitation program	Significant improvements in avoidance and intrusion at 1.5 months, and in emotional reactivity at 4.3 months, *P*<.05) in SC participants, whereas CAU participants experience deterioration of these symptoms; Reduction of avoidance behaviors in SC participants was sustained for up to 24 months; Non-significant difference in distress reduction between SC and CAU (*P* > .05)
Jansen et al. (2019) Amsterdam, the Netherlands	Step 1—Watchful waiting (2 weeks) Step 2—Guided self-management via the internet or a booklet (5 weeks) Step 3—Face-to-face problem-solving therapy (6 sessions maximum) Step 4—Specialized psychological therapy or psychotropic medications	Progressive	Mixed	Persistent symptoms (HADS-total > 14, or HADS-A > 7 or HADS > 7)	No psychosocial care for 52 participants; Psychological treatment and/or psychotropic medication for 20 participants	Higher baseline quality of life and functionality scores (1.0-1.1 per point increase) and the absence of psychiatric disorder (OR 4.8, 95% CI=1.3 to 18.2) increased the likelihood of recovery after step 1; Better emotional functioning, lower distress and lower dyspnea increased the likelihood of recovery after step 2; SC was more effective (*P*<.05) for women, those within 1 year of treatment completion, those diagnosed with anxiety or depression, or with worse HADS scores at baseline
Schuurhuizen et al. (2019) the Netherlands	Step 1—Watchful waiting (3 weeks) Step 2—Guided self-management using educational materials (5-7 weeks) Step 3—Problem-solving therapy delivered by a nurse (10 sessions/10 weeks) Step 4—Specialized psychological intervention and/or psychotropic medications	Progressive	Mixed	Elevated distress (HADS score ≥13 or DT/PT score ≥5)	Distress identified on ad hoc basis by oncologists and nurses	No conclusive evidence due to low uptake of the intervention (11.4% of those allocated to the intervention arm); No significant difference in HADS scores (*P* > .05); SC had a positive effect on cognitive functioning and patient satisfaction at 12 months (*P*<.05)
Hauffman et al. (2020) Mid-Sweden	Step 1—Internet-based educational lectures on self-care strategies, access to a peer support discussion form, and nurse support (available from randomization to follow-up end) Step 2—Internet-based CBT program (offered once at 1-, 4-, or 7-months post randomization) guided by weekly support from a psychologist, in addition to all support offered at step 1	Progressive	Internet-based	Persistent symptoms (HADS-A or HADS-D score > 7)	Routine information related to disease and treatments, psychosocial support from nurses and physicians, or additional support from a counselor or the hospital’s church, provided at the patient’s discretion	SC resulted in a statistically significant reduction in adjusted mean depression levels (−0.5, 95% CI=−1.1 to −0.0, *P*<.05), but this did not reach clinically meaningful change (<20%); No significant differences between SC and CAU were observed for all other symptoms; A larger proportion of SC participants reported a clinically meaningful decrease ( > 2 points) in depression levels (54% vs 35%) and anxiety (52% vs 38%) at 10 months
Lynch et al. (2020) Melbourne Australia	Step 1—Treatment as usual Step 2—Guided self-management using a psychoeducational self-management booklet, and a telephone support session with a clinical or research psychologist Step 3—Five individual therapy sessions with a trained clinical psychologist	Stratified	Mixed	Persistent symptoms Step 1 - > 2 (FCRI-SF was 13-21 or FoP-Q-SF score was 24-33); Step 2 - > 3 (FCRI-SF score ≥22 or FoP-Q-SF score ≥34)	Standard monitoring from their primary care medical and nursing staff	Lower FCR symptoms were observed in 62% (13 out 21) of cases after step 2; in 80% (8 out of 10) of cases who read ≥75% of the booklet; and in 71% (5 out 7) of cases post the individual therapy sessions
Zhou et al. (2020) Boston, United States	Step 1—One-hour group sleep education session delivered by a clinical psychologist Step 2—Three group-based CBT-Insomnia (CBT-I) sessions, plus a self-guided instructional workbook	Progressive	In-person	Persistent symptoms Step 1 - > 2 (ISI ≥ 12 after 4 weeks of commencing step 1 intervention); Step 1 - > 2 (ISI <12 after 4 weeks of step 1 interventions but increased to ≥12 after 8 or 12 weeks of step 1 intervention)	No control group	The mean ISI scores significantly improved (P<.05) after step 1 (baseline: 17.1; week 4: 11.2; week 8: 6.9, week 12: 6.1) and step 2 (baseline: 16.9; week 4: 8.8; week 8: 9.5); Mood disturbance significantly improved (*P*<.05) after step 2 intervention (POMS-SF score: baseline: 15.8; week 8: 7.7)
Savard et al. (2021) Quebec, Canada	Step 1—Internet-based, CBT-I training using educational videos and written treatment materials, for cases with an ISI score ≥8 and <15. Participants with an ISI score ≥15 received 6 weekly CBT-I sessions administered individually by a clinician Step 2—Three 50-minute booster sessions of CBT-I, administered individually by a clinician every 2 weeks	Stratified	Mixed	Persistent insomnia symptoms (ISI≥8 or intake of hypnotic medication ≥1 night/week)	Six weekly sessions of CBT-I administered by a clinician, with self-directed reading of treatment materials completed beforehand	SC was clinically noninferior; SC showed significant improvements over time in sleep medication usage, fatigue, distress symptoms and HRQoL measures (time effects: *P*<.05), but no significant differences were observed between the intervention arms
Schutte et al. (2021) Amsterdam, the Netherlands	Step 1—Watchful waiting (2 weeks) Step 2—Guided self-management via the internet or a booklet (5 weeks) Step 3—Face-to-face problem-solving therapy (6 sessions maximum) Step 4—Specialized psychological therapy or psychotropic medications	Progressive	Mixed	Persistent symptoms after each step (HADS score > 7)	No psychosocial care for 52 participants; Psychological treatment and/or psychotropic medication for 20 participants	No differences in sexual interest and enjoyment were observed over 12 months among head and neck cancer patients, *P* > .05; data for lung cancer patients were not available
Borrayo et al. (2023) Colorado, United States	Step 1—Watchful waiting Step 2—Face-to-face orientation and guided self-help strategies Step 3—Problem-solving therapy on coping skills (2 sessions), plus all step 2 components Step 4—CBT (4 sessions), plus all previous components. Unresolved cases were referred to more specialized treatment	Stratified	In-person	Persistent symptoms Step 1: mild PROMIS score 50-59; Step 2: moderate PROMIS score 60-69; Step 3: severe PROMIS score > 70	Usual care, and enhanced care materials (including online and printed materials, and a list of local support groups and mental healthcare providers)	Significant differences in mean scores at 6 months for depression (Δ 1.8, 95% CI=0.5 to 3.0), coping self-efficacy (Δ −15.2, 95% CI=−26.1 to −4.4), emotional distress (Δ 2.0, 95% CI=0.7 to 3.5), and HRQoL (Δ -4.2, 95% CI=−7.5 to −0.9), *P*≤.05; Non-significant difference in mean anxiety scores at 6 months (Δ 1.5, 95% CI=0.2 to 2.7, at 0.01 significance level)
Diggens et al. (2023) Melbourne, Australia	Step 1—Guided self-management CBT-I resource. Low risk participants: 5 weeks reading materials, 1 face-to-face consultation, and a telephone follow-up with a clinician. High-risk participants: referred to specialized sleep service or received CBT-I if declined referral Step 2—Face-to-face CBT-I (4 group or individual sessions delivered weekly or fortnightly by a psychologist)	Stratified	In-person	Persistent insomnia symptoms (ISI score > 7)	No control group	ISI score <8, in 44% of participants (*n* =18) after step 1 (mean score: baseline 15.2 vs postintervention 9.9, *P*<.05); ISI score <8, in 80% of participants (*n*=4) after step 2 (mean score: after step 1=17.4 vs end of group CBT-I=6.2, *P*<.05)
Igelstrom et al. (2023) Mid-Sweden	Step 1—Internet-based educational lectures on self-care strategies, access to a peer support discussion forum, and nurse support (offered from randomization to the end of follow-up) Step 2—Internet-based CBT (offered once at 1, 4, and 7 months, plus all support offered at step 1	Progressive	Internet-based	Persistent symptoms (HADS-A or HADS-D score > 7)	Routine information related to disease and treatments, psychosocial support from the nurses and physicians, or additional support from a counselor or the hospital’s church, provided at the patient’s discretion	Significant reduction in depression scores in SC group at 18 months (small effect = Cohen’s *d* <0.3), but this effect was not observed at 24 months or in complete case analysis (*P* > .05); Statistically non-significant differences in anxiety reduction (Cohen’s *d* <0.2) observed at 18 and 24 months; however, complete case analysis identified a clinically relevant effect at both time points ( > 2-point reductions); Insomnia and fatigue improved similarly in SC and CAU groups at 18 and 24 months, with no significant differences between groups
Williams et al. (2023) Melbourne, Australia	Step 1—Guided self-management (using readings and activities developed by multidisciplinary experts) with weekly online or telephone support from a psychologist (5-7 weeks) Step 2—Internet-based CBT group therapy (4 tailored sessions), plus all step 1 interventions (4 weeks)	Progressive	Internet-based	Persistent symptoms (FACIT-Fatigue score <34 or did not change by ≥10)	No control group	Step 1 significantly improved fatigue levels (3-17 points change in FACIT-F score; *t*(16) 3.7, medium effect Cohen’s *d*=0.6, *P*<.05), compared with baseline; Fatigue improvements were sustained continued at 12 weeks for step 1 and step 2 participants; EQ-5D-5L scores improved after step 1, but this change was not statistically significant (−1.5, *P* > .05)
Temel et al. (2024) Boston, Philadelphia, and Durham, United States	Step 1—Participants received a palliative care visit within 4 weeks of enrollment, followed by ad hoc visits only in the event of a cancer medication change or inpatient admission Step 2—Participants attended monthly appointments with a palliative care clinician, and were examined by the palliative care team during every hospital admission	Progressive	Mixed	A decline in FACT-L score of ≥10	Early palliative care—participants were scheduled for monthly palliative care visits, and were examined by the inpatient palliative care team during every hospital admission	FACT-L scores at 6 months were non-inferior (adjusted mean 100.6 vs 97.8, difference of 2.9, *P*<.05); Mean count of palliative care visits was lower at 6 months (2.4 vs 4.7, adjusted mean difference −2.3, 95% CI=−2.7 to −1.8, *P*<.05) and at 12 months (3.8 vs 7.7, adjusted mean difference -3.9, 95% CI=−4.7 to −3.1, *P*<.05); Shorter hospice length of stay (19.5 vs 34.6 days, adjusted mean difference −15.2 days, noninferiority *P* > .05)

Cost-effectiveness of steppedcare						
Study (year) location	Steppedcare (SC) intervention				Care as usual (CAU)	Effect of SC compared with CAU
	Number and components of SC interventions	SC approach	Delivery mode	Step-up criteria		
Jansen et al. (2017) Amsterdam, the Netherlands	Step 1— Watchful waiting (2 weeks) Step 2—Guided self-management via the internet or a booklet (5 weeks) Step 3—Face-to-face problem-solving therapy (6 sessions maximum) Step 4—Specialized psychological therapy or psychotropic medications	Progressive	Mixed	Persistent symptoms (HADS-A score > 7 and/or HADS-D score > 7)	No psychosocial care for 52 participants; Psychological treatment and/or psychotropic medication for 20 participants	Lower mean cumulative costs (€3950, *P*<.05) and higher QALYs (incremental effect 0.12, 0.05) in the SC group; Bootstrapped cost-utility pairs showed that SC has a 96% probability of producing more effective outcomes at lower costs
Alili et al. (2020) the Netherlands	Step 1—Watchful waiting Step 2—Guided self-management using educational materials Step 3—Problem-solving therapy delivered by a nurse Step 4—Specialized psychological intervention, psychotropic medication, or referral to other support services	Progressive	Mixed	Elevated psychological distress (HADS score ≥13 or DT/PT score ≥5)	Support for psychological distress was provided when identified by a health-care provider	Statistically non-significant differences in total health care, usual care, and nonhealth care costs (Δ –€295, 95% CI=–3844 to 2579; Δ –€857; 95% CI=–2427 to 615; Δ –€1152, 95% CI=–5058 to 2214, respectively); SC showed dominance for HADS and QALYs, with incremental cost-effectiveness ratio of €19 991 saved per HADS point improvement; Statistically non-significant difference in QALYs (*P* > .05); SC probability of cost-effectiveness for QALYs reached 64% at willingness-to-pay of €0 and 79% at €20 000 per QALY
Pilehvari et al. (2024) Boston, United States	Step 1—A 1-hour group-based sleep education session delivered by a clinical psychologist Step 2—Three 1-hour group sessions of CBT-I delivered by clinician, supplemented with a self-guided instructional workbook	Progressive	In-person	Persistent symptoms Step 1- > 2 (ISI ≥ 12 after 4 weeks of commencing step 1 intervention); Step 2- > 3 (ISI <12 after 4 weeks of step 1 interventions but increased to ≥12 after 8 or 12 weeks of step 1 intervention)	No control group	At the end of the trial, 74.5% of participants in the SC group experienced remission of insomnia symptoms, compared with 39.1% in the 3-session CBT-I program (*P*<.05); Probability of cost-effectiveness was 98% in the SC group vs 2% in the 3-session CBT-I program, based on a willingness-to-pay threshold of $480 per percentage of remitted participants
Steel et al. (2024) Pittsburgh, Pennsylvania, United States	Step 1—Facilitated once-weekly CBT (8-12 sessions), delivered by a coordinator over the phone or via video conferencing, depending on the participants’ baseline symptoms and treatment preference Step 2—Psychotherapy, pharmacotherapy, or additional treatment, if clinically indicated	Progressive	Mixed	Persistent symptoms above the clinical range at 12 weeks of the intervention (depression: CES-D score ≥16, ≥3 for pain or fatigue on a 0–10-point scale)	Enhanced CAU: participants were screened for depression, fatigue, and pain. Symptom education and suicidality assessment were provided. Referral options were often based on their treatment preference. Participants with a CES-D score ≥16 or a pain indicator > 5 on a 0-10 scale were referred to a community-based mental health professional or their general practitioner	SC had a greater effect on HRQoL score than CAU (Cohen’s *d*=.09, *P*<.05 for difference); At 6 months, SC showed greater improvements in emotional, functional and physical well-being scores (*P*<.05); Shorter hospital stays and fewer emergency visits contributed to annual per-patient costs that were $17 085 lower in the SC group than CAU; Reduction in symptoms of depression, pain, and fatigue were maintained at 12 months (Cohen’s *d*’=0.15)

Abbreviations: BPI = Brief Pain Inventory; CBT = cognitive behavioral therapy; CES-D = Centre for Epidemiological Studies-Depression; DT/PT = distress thermometer/problem list; EORTC QLQ-H&N35 = European Organisation for Research and Treatment of Cancer Quality of Life Questionnaire Head and Neck-35; EQ-5D-5L = European Quality of Life 5 Dimensions 5 Level Version; FACIT-Fatigue = Functional Assessment of Chronic Illness Therapy-Fatigue; FACT-General = Functional Assessment of Cancer Therapy—General; FCRI-SF = Fear of Cancer Recurrence Inventory-Short Form; FoP-Q-SF = Fear-of-Progression Questionnaire-Short Form; GAD-7 = Generalised Anxiety Screener 7; HADS = Hospital Anxiety and Depression Scale (HADS-A: anxiety, HADS-D: depression); HNC = head and neck cancer; HRQoL  = health-related quality of life; iCBT = internet-based CBT; ISI = Insomnia Severity Index; PHQ‐9 = Patient Health Questionnaire Short Form; POMS-SF = Profile of Mood States-Short Form; PROMIS = Patient-Reported Outcomes Measurement Information System; QALYs = quality-adjusted life years; QoL = quality of life.

### Characteristics of stepped-care interventions and standard care

The structure and content of stepped-care interventions varied across studies (Table 2). Among the 16 distinct RCTs, 9 examined outcomes in a 2-step intervention,^40^^,^^43^^,^^45^^,^^50^^,^^54^^,^^56^^,^^57^^,^^60^^,^^61^ 4 investigated a 3-step intervention,^42^^,^^49^^,^^53^^,^^55^ and 3 focused on a 4-step intervention.^41^^,^^48^^,^^52^ Ten studies administered the progressive stepped approach^40^^,^^45^^,^^48^^,^^52-54^^,^^56^^,^^57^^,^^60^^,^^61^ and 6 administered the stratified approach.^41-43^^,^^49^^,^^50^^,^^55^ Predetermined clinical indicators of symptom progression or persistence were used to assess the effect of therapy at the end of each step (progressive approach) or the assigned step (stratified approach). These indicators were used to determine the next step of the intervention. The least intensive steps typically involved watchful waiting (ie, disease monitoring through periodic assessments)^41^^,^^42^^,^^48^^,^^52^ and guided self-management using expert-developed printed and online materials, such as booklets, problem-solving activities, and cognitive behavioral strategies.^40-43^^,^^45^^,^^48-50^^,^^52^^,^^54-56^ The most intensive step across all studies involved face-to-face consultations with a specialist. One study examined the intervention’s effectiveness in identifying individuals needing symptom management by conducting first-step screening, followed by second-step routine clinical consultation, which facilitated referral to appropriate health-care services (step 3).^53^ Six (out of 22) studies assessed primary and secondary outcomes using the original intervention components.^44^^,^^46^^,^^47^^,^^51^^,^^58^^,^^59^ Participants randomized to CAU received ad hoc emotional support from a medical professional (including a hematologist, oncologist, nurse, or family doctor) or through a standard consultation without a structured psychological intervention, except for those requiring emotional support.^47^^,^^48^^,^^51^^,^^60^ The reasons for dropout during the intervention included death,^54^ self-efficacy for symptom management,^42^^,^^48^ death,^41^^,^^45^^,^^52^^,^^53^^,^^55^^,^^60^^,^^61^ increase in the burden of illness,^42^^,^^43^^,^^48^^,^^49^^,^^52^^,^^53^^,^^55^^,^^56^ accessibility barrier,^42^^,^^48^^,^^50^ and lack of time.^48^^,^^50^^,^^56^

### Efficacy of components of stepped-care interventions

Overall, 8 studies (out of 16) reported improvements in symptoms following participation in guided self-management interventions.^40^^,^^43^^,^^45^^,^^48-50^^,^^56^^,^^57^ One study did not identify any improvement in symptoms,^52^ while 4 did not report data on the improvements associated with self-management interventions.^41^^,^^42^^,^^54^^,^^55^ The overall proportion of participants who experienced an improvement in symptoms following low-intensity therapy (predominately watchful waiting and guided self-help educational materials) ranged from 34%^48^ to 100%.^45^ One study used data from an existing RCT^48^ to examine differences in the effect of the stepped intervention among the subgroups of the participants.^47^ This study revealed that patients were more likely to recover after low-intensity therapy if they had a psychiatric diagnosis (OR = 4.8, 95% CI = 1.3 to 18.2), a higher baseline EORTC QLQ-C30 score (OR range = 1.0-1.1 per point increase), or better scores per point increase in emotional functioning (OR = 1.0, 95% CI = 1.0 to 1.0), dyspnea (OR = 1.0, 95% CI = 1.0 to 1.0), and distress (OR = 0.8, 95% CI = 0.7 to 1.0).^47^ Seven studies (out of 16) incorporated self-administered and facilitated cognitive behavioral therapy (CBT) as moderate- and high-intensity therapy.^41^^,^^43^^,^^45^^,^^50^^,^^54^^,^^56^^,^^57^ The proportions of patients who experienced symptom improvement following CBT, as reported in different studies, were 66%,^50^ 80%,^43^ and 100%.^45^^,^^57^ Problem-solving therapy was incorporated as a moderate-intensity therapy and delivered by a trained expert in 3 studies (out of 16).^41^^,^^48^^,^^52^ However, the proportion of patients who received this therapy was low in all studies.^41^^,^^48^^,^^52^

### Effect of stepped-care interventions on symptom severity

#### Psychological distress

Eleven studies assessed the effect of stepped-care on psychological distress^40-42^^,^^45^^,^^48^^,^^50^^,^^52-55^^,^  ^58^ using primarily the HADS. Of these 11 studies, 5 reported an overall positive effect of stepped-care, 4 found no significant differences, and 2 yielded inconclusive results.^42^^,^^52^ These 5 studies identified a positive effect on distress among stepped-care participants compared with CAU participants.^41^^,^^45^^,^^48^^,^^54^^,^^58^ The interventions comprised either 2 steps (including guided self-management strategies and ad hoc support from a trained professional)^45^^,^^54^^,^^58^ or 4 steps (including watchful waiting, guided self-management strategies, face-to-face problem solving, specialized psychological therapy and/or medication, or CBT sessions)^41^^,^^48^ over varying observation periods (including 6,^41^^,^^54^ 10,^45^ 12,^48^ and 24 months^58^). The studies reported various indicators of improvement in symptoms in response to stepped-care, including medium to large effect estimates (0.7^54^ and 0.6^48^), a larger than 20% decrease in mean HADS score,^45^ a significant difference in mean depression scores (1.8, *P* < .01),^41^ and a clinically significant decrease in depression scores (>2).^58^ One RCT assessed the sustainability of the observed clinical effects of SC on distress^48^ beyond 12 months.^58^ This RCT identified a sustained improvement in depression scores at 18 months but not at 24 months, while a clinically meaningful improvement in anxiety was observed at both 18 and 24 months.^58^ Two studies reported the proportion of stepped-care participants with a positive response to low-intensity therapy: 34% of participants with distress^48^ and 100% of participants with depression.^45^ For moderate- to high-intensity therapy, the proportions were 26% for participants with distress^48^ and 100% for those with depression.^45^ Four studies demonstrated no significant differences in distress severity scores between participants in the stepped-care and CAU groups.^40^^,^^45^^,^^53^^,^^55^ These studies also reported several other benefits of stepped interventions, including decreased cancer-related stress reactions,^40^ greater self-reported reduction in depression and anxiety,^45^ enhanced identification of patients with unmet support needs,^53^ and reduced symptoms among participants with higher baseline distress scores (HADS≥15) and those with disease progression.^55^ Two studies with inconclusive findings reported low uptake and adherence to stepped-care interventions.^42^^,^^52^ Depression symptoms were also assessed using the Centre for Epidemiological Studies—Depression Scale, which showed improvement in scores among the intervention’s participants at 6 and 24 months of the observation period.^60^

#### Insomnia

Six studies assessed the effect of stepped-care on alleviating insomnia symptoms.^43^^,^^45^^,^^50^^,^^57-59^ In all 6 studies, the intervention comprised 2 steps, generally involving guided self-management (educational materials or CBT resources) and a CBT program delivered by a trained psychologist or a behavioral sleep medicine clinician.^43^^,^^45^^,^^50^^,^^57-59^ In 4 studies, the interventions primarily targeted insomnia symptoms in participants with sleeping difficulties (ie, ISI scores indicating subthreshold, moderate, or severe symptoms).^43^^,^^50^^,^^57^^,^^59^ These studies demonstrated improvement in sleep difficulties with ISI severity scores decreasing from 15.2-17.1 at baseline^43^^,^^50^^,^^57^ to 6.2 at 2.1 months,^43^ 6.9 at 12 months,^50^ and 9.5 at 18 months (for participants with delayed entry into step 2).^57^ Three of these 4 studies assessed the effect of stepped-care using a single-arm, non-randomized design.^43^^,^^57^^,^^59^ At the end of step 1, the remission rates observed in the intervention group were 44%,^43^ 51%,^57^^,^^59^ and 58%.^50^ Remitted participants had a shorter duration and lower severity of insomnia symptoms compared with nonremitted participants (*P* < .05).^57^ At the end of step 2, the reported remission rates in the intervention group were 66%^50^ and 86%.^57^ Detailed information about the characteristics of remitted patients was not reported in these 2 studies^50^^,^^57^; however, the desire to receive professional support was common among those who participated in step 2.^57^ In the Diggens et al. trial, the participation of individuals referred to a high-intensity intervention was low (5 out of 17 participants) due to other health demands, insufficient time, and alternative support, which precluded efficacy assessment.^43^ Two studies assessed the effect of a 2-step, distress-targeted stepped-care intervention on insomnia symptoms in the same participant sample over a 12-month observation period,^45^ with follow-up assessments at 18 and 24 months.^58^ These studies did not identify significant differences in insomnia symptoms between participants in the intervention and CAU arms.^45^^,^^58^ The risk of bias in 2 non-randomized studies was moderate^43^^,^^57^; however, the findings were consistent with those reported in RCTs showing a low risk of bias.^45^^,^^50^^,^^58^

#### Fatigue

Five studies assessed the effectiveness of stepped interventions in reducing fatigue.^45^^,^^50^^,^^54^^,^^56^^,^^60^ Two trials primarily focused on participants experiencing fatigue symptoms,^54^^,^^56^ where the interventions consisted of guided self-management support in step 1 and an internet-based CBT in step 2. These trials demonstrated a reduction in fatigue levels at 3 (0.6)^56^ and 6 (effect size 0.3)^54^ months of the intervention. The observed reduction in fatigue was sustained at 12 months.^56^ The effect of stepped interventions on fatigue was also assessed in another 3 studies, which provided strategies that primarily targeted psychological distress,^45^ insomnia,^50^ and fatigue.^60^ Distress-targeted therapy led to a moderately clinically relevant change in baseline fatigue score (assessed using the Functional Assessment of Chronic Illness Therapy Fatigue Subscale) at 10 months of observation; however, this change was statistically non-significant.^45^ Conversely, insomnia-focused therapy resulted in a significant improvement in fatigue (effect size = 0.9).^50^ Furthermore, therapy targeting QoL was associated with a more substantial reduction in fatigue scores in the stepped intervention group compared with those receiving CAU.^60^ Overall, the risk of bias was classified as low across the RCTs that assessed fatigue.^45^^,^^50^^,^^54^^,^^60^

#### HRQoL issues

Eight studies assessed the effectiveness of stepped-care in improving HRQoL, primarily as a secondary outcome.^41^^,^^45^^,^^48^^,^^50^^,^^54^^,^^56^^,^^60^^,^^61^ Improvement in HRQoL was observed across the enrollment period in 3 studies that compared changes in HRQoL score in the intervention participants compared with CAU (−4.2, 95% CI = −7.5 to −0.9, *P* < .05^41^; >10 points difference, *P* < .05^48^; −2.2, *P* = .05).^54^ Insomnia-focused CBT showed comparable effects in enhancing HRQoL for participants in the CAU and stepped-care arms (*F* [4589] = 1.1, *P* = .4), with small to moderate effect sizes observed at 6 weeks (intervention Cohen’s *d* = 0.5; CAU Cohen’s *d* = 0.7).^50^ However, no significant time or treatment effects were observed after 6 weeks of the intervention (*F* [4589] = 1.1, *P* = .4; Cohen’s *d* = 0.2).^50^ A similar transient improvement in HRQoL was reported in a feasibility study of a fatigue-focused stepped-care CBT trial, which identified an improvement in scores from 7.5 at baseline to 5.5 at 6 weeks (ie, after step 1). However, this improvement declined to 6.5 after 6 weeks of the intervention.^56^ A distress-focused, internet-based RCT identified non-significant clinical differences between the treatment arms on the overall global health status (defined on the EORTC QLQ-C30 scale).^45^ However, small clinical differences in social functioning, pain, dyspnea, insomnia, and diarrhea were observed in the intervention group.^45^ Small but non-significant improvements in HRQoL scores were also reported in another study.^60^ In palliative care settings, the stepped care intervention demonstrated both statistically and clinically non-inferior effects on quality of life compared to the early palliative care program at the end of the intervention (adjusted mean FACT-L score: 100.6 vs 97.8; *P* < .05).^61^

#### Fear of cancer recurrence

Fear of cancer recurrence (FCR) was assessed in one non-randomized study that assigned survivors of metastatic melanoma with sub-threshold FCR to self-management strategies (≈44% of 61), and those with moderate to severe symptoms (≈20% of 61) to individual therapy sessions.^49^ The self-management strategies had a small effect on FCR (Cohen’s *d* = 0.1), while the 5 individual therapy sessions had larger effects (Cohen’s *d* = 0.7).^49^ The mean scores indicating changes in FCR and fear of progression (FoP) in response to the interventions were calculated using the FCR Inventory Short Form (FCRI-SF) and FoP Questionnaire Short Form (FoP-Q-SF) questionnaires. The findings showed a higher effect of the individual therapy sessions (FCRI-SF = 24.3 vs 20.6; FoP-Q-SF = 37.3 vs 33.7) than self-management (FCRI-SF = 17.7 vs 16.9; FoP-Q-SF = 29.1 vs 29.0).^49^

#### Sexual health

The efficacy of the intervention in reducing unmet sexual health needs was examined in 2 studies that administered interventions targeting psychological distress.^51^^,^^55^ After adjusting for baseline differences in sexual interest and enjoyment, the 4-step distress-focused intervention had a non-significant effect on symptom reductions compared with CAU at any step (*P* = .9).^51^ The presence of psychological distress (*P* = .6) or unmet sexual needs (*P* = .6) did not moderate the change in symptoms.^51^ Supporting evidence of a non-significant effect on symptoms of sexuality was observed in the second study, which identified a minimal difference between stepped-care intervention and CAU (adjusted difference = 0.2, 95% CI = −0.8 to 1.3, *P* = .7).^55^

### Comparative cost-effectiveness

Four studies examined the cost-effectiveness of stepped-care interventions^44^^,^^46^^,^^59^^,^^60^ primarily using randomized designs.^44^^,^^46^^,^^60^ These interventions targeted psychological distress^44^^,^^46^ and HRQoL issues^60^ in cancer patients, as well as insomnia symptoms in cancer survivors.^59^ Evidence on the cost-effectiveness of the distress-focused CAU and stepped interventions was inconsistent.^44^^,^^46^ One study estimated a statistically significant increase in the number of QALYs gained (incremental effects = 0.12) and cost savings (mean cumulative costs = €3950) in the stepped-care group compared with CAU.^46^ Another study estimated non-significant differences between the 2 intervention groups in total health-care costs (−€295; 95% CI = –3844 to 2579) and QALYs (mean difference 0.0, 95% CI = –0.0 to 0.1).^44^ However, a potential cost savings of €19 991 for each point improvement on the HADS was associated with the intervention.^44^ The integration of screening and stepped collaborative interventions was also linked to a $17 085 reduction in the annual per-patient costs of admissions and readmissions to inpatient wards and emergency departments, compared with CAU.^60^ Incremental cost-effectiveness analyses of insomnia-focused stepped intervention estimated an additional $173.0 (95% CI = 147.8 to 198.19) for every percentage point increase in the proportion of survivors with remission of symptoms, compared with CAU.^59^

## Discussion

This review synthesized evidence from 17 RCTs and 5 non-randomized experimental studies conducted in the past 8 years to evaluate the efficacy and cost-effectiveness of stepped-care in managing cancer-related symptoms.^40-61^ While most studies exhibited a low risk of bias, a few showed concerns related to the randomization domain.^40^^,^^48^ Several studies reported evidence supporting the potential benefits of integrating stepped-care into routine clinical services during cancer treatments and the post survival phase. This integration may help reduce the severity of symptoms,^41^^,^^43^^,^^48-50^^,^^54^^,^^57-61^ improve patients’ social functioning and cognitive capacity,^52^ enhance referral to psychosocial services,^53^ decrease visits to palliative care,^61^ and lessen adverse emotional reactions during anticancer treatment.^40^ These positive outcomes were observed in comparison to CAU and across varying intensities of the stepped interventions.^43^^,^^49^^,^^56^^,^^57^ There were no pronounced differences in the outcomes based on the stepped approach (stratified vs progressive) or according to the number of steps; however, targeted and facilitated therapy appeared to (beneficially) moderate the effect of the intervention on symptom reduction through tailored therapeutic support and improved adherence to therapy. The response to stepped interventions varied across the studies, which can be attributed to methodological differences (including stage and prognosis of cancer, therapy components, severity of symptoms, and duration of intervention) and regional differences in the availability and quality of CAU.^26^ Overall, the intervention uptake was generally low and precluded the formulation of conclusive recommendations in 2 trials.^42^^,^^52^ Nevertheless, the potential utility of the intervention was evident in several studies with smaller samples,^56^^,^^57^ warranting further investigations with larger samples.

The reviewed RCTs primarily focused on improving psychological distress symptoms, either alone or in conjunction with fatigue, chronic pain, unmet sexual needs, emotional reactivity, insomnia, and low QoL. Across the studies, the improvement in distress was determined based on a HADS cutoff score greater than 7, which provides an optimal detection of distress changes when evaluating cancer patients.^62^^,^^63^ Several trials highlighted the beneficial effect of stepped-care on distress reduction.^41^^,^^42^^,^^50^^,^^54^ Evidence of improved uptake of support for psychiatric morbidity upon integrating recommended screening and stepped-care^60^ highlights the added value of implementing the model to enable better access to personalized care for patients with and without high symptom severity scores. The organizational benefits associated with stepped-care could help address current challenges in mental health-care, such as the widespread gap between the need for treatment and the provision of care.^64-66^ As for the trials that reported limited or no effect,^40^^,^^45^^,^^53^^,^^55^ the findings were influenced by limitations in study design that warrant modifications in future studies. Specifically, the necessary improvements include extending trial durations to generate conclusive evidence for individuals with severe symptoms,^53^^,^^55^ implementing effective strategies to mitigate dropout in the intervention group,^53^ and increasing sample sizes to better evaluate treatment responses and response rates across diverse cancer populations.^40^ It is also important to utilize objective methods to ascertain psychological distress at baseline,^40^ such as diagnostic interviews and assessment of psychological and physiological indicators.^67^ In addition, increasing the number of participating sites can help minimize the effect of attrition on the proportion of assessed participants.^45^^,^^55^

All insomnia-targeted interventions contributed to improvements in symptoms,^43^^,^^50^^,^^57^ and notably, 2 RCTs identified clinically meaningful changes in fatigue symptoms among cancer patients managed using CBT strategies targeting insomnia and distress.^45^^,^^50^ This suggests that implementing stepped interventions which incorporate guided CBT (delivered in-person or through digital health platforms) could enhance the efficiency of care delivery. This can, in turn, reduce the adverse effects of insomnia on psychological functioning.^68-71^ Evidence on the efficacy of stepped interventions for managing fear of recurrence^49^ and unmet sexual needs^51^^,^^55^ in cancer survivors and patients is limited. Further research is needed to assess symptom-focused stepped interventions to evaluate their effectiveness in reducing fear of recurrence and addressing sexual needs in cancer patients and survivors.

The cost-effectiveness analyses showed that stepped interventions for newly diagnosed cancer patients^46^^,^^60^ and breast cancer survivors^59^ could reduce the cost of symptom management by optimizing the use of resources, while also contributing to improved QALYs,^46^ insomnia symptoms,^59^ and HRQoL.^60^ Inconsistent evidence of cost savings and symptom improvement was observed in patients with metastatic colorectal cancer, which can be explained by the complexity of psychological needs^72^ and the low uptake of the intervention among these patients. Additionally, the impact of a metastatic diagnosis may lead to a higher likelihood of loss to follow-up due to death,^73^ as well as differences in HADS cutoff used to step-up patients with depression or anxiety (mild^46^ vs moderate).^44^ Moreover, many cancer patients receive emotional support through family members and primary care.^74^

The incorporation of evidence-based therapeutic approaches is a key driver of the effectiveness of the reviewed stepped interventions. Self-management strategies are considered low-intensity interventions that enable individuals with medical and psychological complexity to self-manage their needs using guided support.^75^ In this review, 8 studies reported a positive response and clinically meaningful reduction in symptoms (namely psychological distress, fear of cancer recurrence, insomnia, and fatigue) following guided self-administered support,^40^^,^^43^^,^^45^^,^^48-50^^,^^56^^,^^57^ which indicates that more individuals can be managed with less intensive therapy. The effectiveness of self-management interventions (incorporating individual or group delivery) has been reported in a previous systematic review^76^ as an effective way to improve QoL indicators in cancer survivors. Supporting evidence of self-management effectiveness was also reported in a second systematic review that focused on managing distress in adult cancer patients undergoing anticancer treatments.^77^ Our findings also support the importance of facilitation by nurses, psychologists or clinicians during the self-management step.^40^^,^^43^^,^^45^^,^^48-50^^,^^56^^,^^57^ A systematic review previously reported supporting evidence indicating the importance of professional facilitation when administering self-help interventions.^77^ Four studies illustrated the effectiveness of self-support accessed through digital platforms,^45^^,^^48^^,^^50^^,^^56^ which indicates that stepped-care incorporating self-support can help expedite access to symptom management strategies in settings with high demand for supportive services.^78^ It is important to note that patients with higher severity scores might experience greater benefits from low-intensity therapy if the intervention was offered over a longer duration compared with that offered in the RCT context.^21^

Although facilitated problem-solving therapy focused on adaptive skills has been incorporated in 2 distinct stepped-care studies,^48^^,^^52^ the small number of participants who received this therapy precluded the formulation of conclusive recommendations. Nevertheless, the effectiveness of problem-solving in managing symptoms experienced by cancer^79^ and noncancer^80^^,^^81^ populations has been previously demonstrated. Future investigations on the effectiveness of the guided, self-administered problem-solving therapy component of stepped interventions in cancer treatment centers and survivorship follow-up clinics are warranted.

The reviewed evidence showed that low-intensity internet-based CBT-I was not clinically inferior to CAU^50^ and can help improve sleep quality in cancer patients^50^ and survivors.^43^ Accessing personalized insomnia therapy through internet platforms can improve accessibility, particularly useful in low-resource settings, and may help alleviate the health and economic burdens associated with the high prevalence of insomnia among cancer populations.^68^ Facilitated, internet-based CBT targeting psychological distress,^45^^,^^58^ fatigue,^56^ and multiple comorbid symptoms^54^ has demonstrated small^45^^,^^58^ and medium^54^^,^^56^ treatment effects, which were sustained for short (3-6 months^54^^,^^56^) and long (12-24 months^45^^,^^58^) periods. Overall, the findings indicate that incorporating facilitated internet-based therapy for individuals with persistent symptoms can offer the opportunity to address spatial and temporal constraints. A previous meta-analysis showed that psychotherapy administered by an experienced expert has the greatest effect on cancer patients’ QoL and emotional well-being.^82^ Further research is needed to identify effective strategies to enhance adherence to internet-based CBT among cancer patients^45^^,^^58^ and survivors.^83^

### Clinical practice and future research

Disseminating educational resources about the value of stepped-care to the relevant stakeholders via digital and other tailored avenues can help increase the adoption of interventions and optimize the efficient use of evidence-based practices.^74^^,^^84^ Optimized accessibility to digital platforms with stepped-care resources at key time points (eg, end of the treatment period and during the transition to survivorship care or adult healthcare services) can also help address support needs promptly.

Further research is needed to explore the potential of stepped-care interventions in cancer care settings and address the existing methodological limitations. Assessing the practicalities of implementing stepped-care interventions in publicly funded or privately managed routine clinical settings (eg, demand for clinical care, and acceptability of intervention to diverse patients, and administrative leadership) within the existing cancer care system is warranted. The application of mathematical simulation models with alternative decision parameters (such as patient characteristics, treatment components, intervention duration, and cost estimations) can provide the opportunity to assess the optimal structure of the stepped intervention and the potential health and economic outcomes.^85^^,^^86^ Standardizing evidence-based therapeutic components, outcome measurement scales, and effect estimation methods can help enhance the comparability of evidence collected from cancer subgroups with similar clinical characteristics. The intervention duration was short in most studies (6-12 months), precluding the assessment of outcomes in patients with severe symptoms that require prolonged follow-up. Conducting observational research using clinical records collected through primary care or long-term survivorship clinics could help provide insight into the long-term effect of the stepped intervention.^87^ In addition, while targeted interventions appeared to result in greater symptom reduction, potential synergistic or adverse effects of intervention interactions require further investigation, particularly as many patients experience multiple concurrent symptoms that may necessitate different types of interventions. Further investigations are also warranted to understand the sustainability of improved symptoms in cancer patients and survivors, and the effectiveness of the interventions in improving under-researched symptoms (including fear of cancer recurrence and unmet sexual needs).^49^^,^^51^^,^^55^

The review has three main limitations that should be considered when interpreting its findings. First, heterogeneity in cancer treatment exposures, tumor stage, and access to social support may have influenced the recovery rates observed in the stepped-care intervention group. Second, the total sample size and the low uptake of high-intensity interventions limited the generalizability of the evidence and precluded conclusive recommendations regarding the efficacy of stepped-care. Nevertheless, the benefits identified in the RCTs were consistent with those observed in noncancer populations.^16^^,^^88^ Third, the evidence related to moderators of the intervention effect should be interpreted with caution, as most studies were not powered or specifically designed to examine subgroup differences.

## Conclusion

This systematic review revealed that stepped-care interventions that incorporate evidence-based therapeutic approaches can help reduce cancer-related symptoms, including psychological distress, insomnia, fatigue, impairments in physical and cognitive function, and low HRQoL. Several RCTs have highlighted the essential role of trained health-care professionals in facilitating participant uptake and continued engagement in supportive services. The observed positive clinical response in participants following guided self-management suggests that this approach can improve access to supportive care, particularly in settings characterized by high demand and limited resources. Digital CBT interventions show promise for increasing access to affordable services. While evidence on cost-effectiveness remains limited, preliminary findings suggest that stepped-care interventions may lead to cost savings, warranting the need for further investigations to confirm these findings in more representative cancer populations. Future research should focus on assessing the long-term effects of stepped-care in real health-care settings and determining the optimal delivery methods for each type of intervention. These efforts will be essential for refining and maximizing the benefits of stepped-care approaches in cancer care.

## Supplementary Material

djaf153_Supplementary_Data

## Data Availability

The data reviewed in this study were extracted from published peer-reviewed studies. No new data were generated or analyzed to draw conclusions or formulate recommendations.

## References

[1] Chan RJ , Gordon LG, Tan CJ, et al Relationships between financial toxicity and symptom burden in cancer survivors: a systematic review. J Pain Symptom Manage. 2019;57:646-660.e1. 30550833 10.1016/j.jpainsymman.2018.12.003

[2] Lustberg MB , Kuderer NM, Desai A, Bergerot C, Lyman GH. Mitigating long-term and delayed adverse events associated with cancer treatment: implications for survivorship. Nat Rev Clin Oncol. 2023;20:527-542. 37231127 10.1038/s41571-023-00776-9 PMC10211308

[3] Thong MSY , van Noorden CJF, Steindorf K, Arndt V. Cancer-related fatigue: causes and current treatment options. Curr Treat Options Oncol. 2020;21:17-17. 32025928 10.1007/s11864-020-0707-5 PMC8660748

[4] Shin H , Dudley WN, Bhakta N, et al Associations of symptom clusters and health outcomes in adult survivors of childhood cancer: a report from the St Jude lifetime cohort study. J Clin Oncol. 2023;41:497-507. 36166720 10.1200/JCO.22.00361 PMC9870227

[5] Dantzer R , Meagher MW, Cleeland CS. Translational approaches to treatment-induced symptoms in cancer patients. Nat Rev Clin Oncol. 2012;9:414-426. 22641361 10.1038/nrclinonc.2012.88 PMC3412618

[6] Feuerstein M , Bruns GL, Pollman C, Todd BL. Management of unexplained symptoms in survivors of cancer. J Oncol Pract. 2010;6:308-311. 21358961 10.1200/JOP.2010.000088 PMC2988665

[7] Yannitsos D , Qi S, Davies O, Watson L, Barbera L. Trends in symptom severity and complexity in patients undergoing radiation therapy. BMC Cancer. 2025;25:390-398. 40038635 10.1186/s12885-025-13587-1 PMC11877897

[8] Koo MM , Swann R, McPhail S, et al Presenting symptoms of cancer and stage at diagnosis: evidence from a cross-sectional, population-based study. Lancet Oncol. 2020;21:73-79. 31704137 10.1016/S1470-2045(19)30595-9 PMC6941215

[9] Harrington CB , Hansen JA, Moskowitz M, Todd BL, Feuerstein M. It’s not over when it’s over: long-term symptoms in cancer survivors—a systematic review. Int J Psychiatry Med. 2010;40:163-181. 20848873 10.2190/PM.40.2.c

[10] Fitzmaurice C , Akinyemiju TF, Al Lami FH, et al; Global Burden of Disease Cancer Collaboration. Global, regional, and national cancer incidence, mortality, years of life lost, years lived with disability, and disability-adjusted life-years for 29 cancer groups, 1990 to 2016: a systematic analysis for the Global Burden of Disease Study. JAMA Oncol. 2018;4:1553-1568. 29860482 10.1001/jamaoncol.2018.2706 PMC6248091

[11] Jacobs LAP , Shulman LNP. Follow-up care of cancer survivors: challenges and solutions. Lancet Oncol. 2017;18:e19-e29. 10.1016/S1470-2045(16)30386-2 28049574

[12] Ehrhardt MJ , Krull KR, Bhakta N, et al Improving quality and quantity of life for childhood cancer survivors globally in the twenty-first century. Nat Rev Clin Oncol. 2023;20:678-696. 37488230 10.1038/s41571-023-00802-w

[13] Gunn AH , Sorenson C, Greenup RA. Navigating the high costs of cancer care: opportunities for patient engagement. Future Oncol. 2021;17:3729-3742. 34296620 10.2217/fon-2021-0341

[14] Sullivan RP , Peppercorn JP, Sikora KP, et al Delivering affordable cancer care in high-income countries. Lancet Oncol. 2011;12:933-980. 21958503 10.1016/S1470-2045(11)70141-3

[15] van Kalsbeek RJ , Hudson MM, Mulder RL, et al; International Childhood Cancer Outcome Project Participants. A joint international consensus statement for measuring quality of survival for patients with childhood cancer. Nat Med. 2023;29:1340-1348. 37322119 10.1038/s41591-023-02339-y

[16] Australian Government Department of Health. PHN mental health flexible funding pool programme guidance: stepped care. Canberra: Australian Government Department of Health; 2019.

[17] Richards DA , Bower P, Pagel C, et al Delivering stepped care: an analysis of implementation in routine practice. Implement Sci. 2012;7:3-3. 22248385 10.1186/1748-5908-7-3 PMC3283464

[18] Smith SK , Loscalzo M, Mayer C, Rosenstein DL. Best practices in oncology distress management: beyond the screen. Am Soc Clin Oncol Educ Book. 2018;38:813-821. 30231391 10.1200/EDBK_201307

[19] Sijbrandij M , Kleiboer A, Farooq S. Editorial: low-intensity interventions for psychiatric disorders. Front Psychiatry. 2020;11:619871. 10.3389/fpsyt.2020.619871 33324269 PMC7723924

[20] Psycho-oncology Co-operative Research Group. The Australian clinical pathway for the screening, assessment and management of anxiety and depression in adult cancer patients. Accessed November 30, 2023. https://www.pocog.org.au/doc/ClinicalPathways_Sept%202017.pdf 10.1002/pon.3920 26268799

[21] Bower P , Kontopantelis E, Sutton A, et al Influence of initial severity of depression on effectiveness of low intensity interventions: meta-analysis of individual patient data. BMJ. 2013;346:f540. 23444423 10.1136/bmj.f540 PMC3582703

[22] Nicholas J , Ringland KE, Graham AK, et al Stepping up: predictors of ‘stepping’ within an iCBT stepped-care intervention for depression. Int J Environ Res Public Health. 2019;16:4689. 31775297 10.3390/ijerph16234689 PMC6926538

[23] Ho FYY , Yeung WF, Ng THY, Chan CS. The efficacy and cost-effectiveness of stepped care prevention and treatment for depressive and/or anxiety disorders: a systematic review and meta-analysis. Sci Rep. 2016;6:29281. 10.1038/srep29281 27377429 PMC4932532

[24] Roberts LN , Nixon RDV. Systematic review and meta-analysis of stepped care psychological prevention and treatment approaches for posttraumatic stress disorder. Behav Ther. 2023;54:476-495. 10.1016/j.beth.2022.11.005 37088505

[25] Baglioni C , Espie CA, Altena E, et al Cognitive behavioural therapy for insomnia disorder: extending the stepped care model. J Sleep Res. 2023;32:e14016. 10.1111/jsr.14016 37584390

[26] Andersen BL , Lacchetti C, Ashing K, et al Management of anxiety and depression in adult survivors of cancer: ASCO guideline update. J Clin Oncol. 2023;41:3426-3453. 37075262 10.1200/JCO.23.00293

[27] Smith AB , Girgis A, Taylor N, et al Step-by-step: a clinical pathway for stepped care management of fear of cancer recurrence—results of a three-round online Delphi consensus process with Australian health professionals and researchers. J Cancer Surviv. 2024. 10.1007/s11764-024-01685-1 PMC12988909 39375279

[28] Reeves P , Szewczyk Z, Proudfoot J, Gale N, Nicholas J, Anderson J. Economic evaluations of stepped models of care for depression and anxiety and associated implementation strategies: a review of empiric studies. Int J Integr Care. 2019;19:8. 10.5334/ijic.4157 PMC6588024 31244562

[29] Shaw J , Kamphuis H, Sharpe L, et al Setting an international research agenda for fear of cancer recurrence: an online Delphi consensus study. Front Psychol. 2021;12:596682. 33692719 10.3389/fpsyg.2021.596682 PMC7938308

[30] Pradhan P , Sharpe L, Menzies RE. Towards a stepped care model for managing fear of cancer recurrence or progression in cancer survivors. Cancer Manage Res. 2021;13:8953-8965. 10.2147/CMAR.S294114 PMC8645945 34880676

[31] Abdalla T , Singh G, Roudbaneh S, Sousa M, Serwaa D, Peate M. The efficacy and cost-effectiveness of stepped care models utilised in the management of therapy-related symptoms in cancer patients and survivors: a systematic review. Updated March 20, 2023. Accessed July 10, 2023. https://www.crd.york.ac.uk/prospero/display_record.php?RecordID = 298245&VersionID = 1924123

[32] World Medical Association. WMA Declaration of Helsinki—ethical principles for medical research involving human subjects. Updated September 6, 2022. Accessed February 1, 2024. https://www.frontiersin.org/articles/10.3389/fmed.2024.1360653/full#ref1

[33] Page MJ , McKenzie JE, Bossuyt PM, et al The PRISMA 2020 statement: an updated guideline for reporting systematic reviews. BMJ (Online). 2021;372:71. 10.1136/bmj.n71 PMC8005924 33782057

[34] Frandsen TF , Nielsen MFB, Eriksen MB. Avoiding searching for outcomes called for additional search strategies: a study of Cochrane review searches. J Clin Epidemiol. 2022;149:83-88. 10.1016/j.jclinepi.2022.05.015 35661816

[35] Alharbi A , Stevenson M. Refining Boolean queries to identify relevant studies for systematic review updates. J Am Med Inf Assoc. 2020;27:1658-1666. 10.1093/jamia/ocaa148 PMC7750994 33067630

[36] Leblanc V , Hamroun A, Bentegeac R, Le Guellec B, Lenain R, Chazard E. Added value of medical subject headings terms in search strategies of systematic reviews: comparative study. J Med Internet Res. 2024;26:e53781. 39561364 10.2196/53781 PMC11615561

[37] Lash TL , VanderWeele TJ, Haneuse S, Rothman KJ. Modern Epidemiology. 4th ed. Wolters Kluwer; 2021. 10.1007/s10654-021-00778-w PMC8416883 34216355

[38] Higgins JPT , Thomas J, Chandler J, et al Cochrane Handbook for Systematic Reviews of Interventions. Cochrane Book Series. 2nd ed. Wiley-Blackwell; 2019.

[39] Sterne JAC , Hernán MA, Reeves BC, et al ROBINS-I: a tool for assessing risk of bias in non-randomised studies of interventions. BMJ. 2016;355:i4919. 10.1136/bmj.i4919 27733354 PMC5062054

[40] Arving C , Assmus J, Thormodsen I, Berntsen S, Nordin K. Early rehabilitation of cancer patients—an individual randomized stepped-care stress-management intervention. Psycho-Oncology. 2019;28:301-308. 30408282 10.1002/pon.4940

[41] Borrayo EA , Juarez-Colunga E, Kilbourn K, et al Stepped-care to improve mental health outcomes among underserved patients with lung and head and neck cancer. Psycho-Oncology. 2023;32:1718-1726. 37772984 10.1002/pon.6223

[42] Braamse AMJ , van Meijel B, Visser OJ, et al A randomized clinical trial on the effectiveness of an intervention to treat psychological distress and improve quality of life after autologous stem cell transplantation. Ann Hematol. 2016;95:105-114. 26420062 10.1007/s00277-015-2509-6 PMC4700101

[43] Diggens J , Bullen D, Maccora J, et al Feasibility and efficacy of ‘can-sleep’: effects of a stepped-care approach to cognitive-behavioral therapy for insomnia in cancer. J Cancer Surviv. 2023. 10.1007/s11764-023-01457-3 PMC11814024 37751126

[44] El Alili M , Schuurhuizen C, Braamse AMJ, et al Economic evaluation of a combined screening and stepped-care treatment program targeting psychological distress in patients with metastatic colorectal cancer: a cluster randomized controlled trial. Palliat Med. 2020;34:934-945. 32348700 10.1177/0269216320913463 PMC7787671

[45] Hauffman A , Alfonsson S, Bill-Axelson A, et al Cocreated internet-based stepped care for individuals with cancer and concurrent symptoms of anxiety and depression: results from the U-CARE AdultCan randomized controlled trial. Psycho-Oncology. 2020;29:2012-2018. 32691455 10.1002/pon.5489 PMC7821133

[46] Jansen F , Krebber AM, Coupé VM, et al Cost-utility of stepped care targeting psychological distress in patients with head and neck or lung cancer. J Clin Oncol. 2017;35:314-324. 27918712 10.1200/JCO.2016.68.8739

[47] Jansen F , Lissenberg-Witte BI, Krebber AMH, et al Stepped care targeting psychological distress in head and neck cancer and lung cancer patients: which groups specifically benefit? Secondary analyses of a randomized controlled trial. Support Care Cancer. 2019;27:4543-4553. 30915569 10.1007/s00520-019-04714-3 PMC6825034

[48] Krebber AMH , Jansen F, Witte BI, et al Stepped care targeting psychological distress in head and neck cancer and lung cancer patients: a randomized, controlled trial. Ann Oncol. 2016;27:1754-1760. 27287209 10.1093/annonc/mdw230

[49] Lynch FA , Katona L, Jefford M, et al Feasibility and acceptability of fear-less: a stepped-care program to manage fear of cancer recurrence in people with metastatic melanoma. J Clin Med. 2020;9:2969. 32937942 10.3390/jcm9092969 PMC7565154

[50] Savard J , Ivers H, Savard MH, et al Efficacy of a stepped care approach to deliver cognitive-behavioral therapy for insomnia in cancer patients: a noninferiority randomized controlled trial. Sleep. 2021;44:1. 10.1093/sleep/zsab166 PMC8598200 34228123

[51] Schutte LER , Melissant HC, Jansen F, et al Effect of stepped care on sexual interest and enjoyment in distressed patients with head and neck cancer: a randomized controlled trial. Sex Med. 2021;9:100304. 33460907 10.1016/j.esxm.2020.100304 PMC7930858

[52] Schuurhuizen C , Braamse A, Beekman A, et al Screening and stepped care targeting psychological distress in patients with metastatic colorectal cancer: the TES cluster randomized trial. J Natl Compr Cancer Netw. 2019;17:911-920. 10.6004/jnccn.2019.7285 31390590

[53] Singer S , Danker H, Roick J, et al Effects of stepped psychooncological care on referral to psychosocial services and emotional well-being in cancer patients: a cluster-randomized phase III trial. Psycho-Oncology. 2017;26:1675-1683. 28665542 10.1002/pon.4492

[54] Steel JL , Geller DA, Kim KH, et al Web‐based collaborative care intervention to manage cancer‐related symptoms in the palliative care setting. Cancer. 2016;122:1270-1282. 26970434 10.1002/cncr.29906 PMC4828258

[55] Turner J , Kelly B, Clarke D, et al A tiered multidisciplinary approach to the psychosocial care of adult cancer patients integrated into routine care: the PROMPT study (a cluster-randomised controlled trial). Support Care Cancer. 2017;25:17-26. 27530996 10.1007/s00520-016-3382-0

[56] Williams LK , Ftanou M, Pearson EJ. Virtual delivery of stepped-care cognitive behaviour therapy for cancer related fatigue: a feasibility study. Integr Cancer Ther. 2023;22:15347354231191701. 37571803 10.1177/15347354231191701 PMC10422894

[57] Zhou ES , Michaud AL, Recklitis CJ. Developing efficient and effective behavioral treatment for insomnia in cancer survivors: results of a stepped care trial. Cancer. 2020;126:165-173. 31550051 10.1002/cncr.32509 PMC6906236

[58] Igelstrom H , Carlsson M, Hauffman A, et al Long-term effects on depression and anxiety of an internet-based stepped care intervention for patients with cancer and symptoms of depression and anxiety. The U-CARE AdultCan trial. Internet Interv. 2023;32:100625. 37273929 10.1016/j.invent.2023.100625 PMC10235429

[59] Pilehvari A , Recklitis CJ, Zhou ES, You W. A retrospective cost-effectiveness analysis of different cognitive-behavioral therapy for insomnia intervention delivery approaches in adult cancer survivors. Psycho-Oncology. 2024;33:e6327. 38497829 10.1002/pon.6327

[60] Steel JL , George CJ, Terhorst L III., et al Patient, family caregiver, and economic outcomes of an integrated screening and novel stepped collaborative care intervention in the oncology setting in the USA (CARES): a randomised, parallel, phase 3 trial. Lancet. 2024;403:1351-1361. 38490230 10.1016/S0140-6736(24)00015-1 PMC11556417

[61] Temel JS , Jackson VA, El-Jawahri A, et al Stepped palliative care for patients with advanced lung cancer: a randomized clinical trial. JAMA. 2024;332:471-481. 38824442 10.1001/jama.2024.10398 PMC11145511

[62] Singer S , Kuhnt S, Götze H, et al Hospital anxiety and depression scale cutoff scores for cancer patients in acute care. Br J Cancer. 2009;100:908-912. 19240713 10.1038/sj.bjc.6604952 PMC2661775

[63] Annunziata MA , Muzzatti B, Bidoli E, et al Hospital anxiety and depression scale (HADS) accuracy in cancer patients. Support Care Cancer. 2020;28:3921-3926. 31858249 10.1007/s00520-019-05244-8

[64] The Lancet Regional Health—Western Pacific. The widened gap in mental health services during the pandemic. Lancet Reg Health West Pac. 2021;15:100320. 34786567 10.1016/j.lanwpc.2021.100320 PMC8568701

[65] Moitra M , Santomauro D, Collins PY, et al The global gap in treatment coverage for major depressive disorder in 84 countries from 2000-2019: a systematic review and Bayesian meta-regression analysis. PLoS Med. 2022;19:e1003901. 35167593 10.1371/journal.pmed.1003901 PMC8846511

[66] Ferrari A , Santomauro D, Herrera A, et al Global, regional, and national burden of 12 mental disorders in 204 countries and territories, 1990–2019: a systematic analysis for the Global Burden of Disease Study 2019. Lancet Psychiatry. 2022;9:137-150. 35026139 10.1016/S2215-0366(21)00395-3 PMC8776563

[67] Crosswell AD , Lockwood KG. Best practices for stress measurement: how to measure psychological stress in health research. Health Psychol Open. 2020;7:2055102920933072. 10.1177/2055102920933072 32704379 PMC7359652

[68] Büttner-Teleagă A , Kim Y-T, Osel T, Richter K. Sleep disorders in cancer—a systematic review. Int J Environ Res Public Health. 2021;18:11696. 10.3390/ijerph182111696 34770209 PMC8583058

[69] Aricò D , Raggi A, Ferri R. Cognitive behavioral therapy for insomnia in breast cancer survivors: a review of the literature. Front Psychol. 2016;7:1162. 27536265 10.3389/fpsyg.2016.01162 PMC4971442

[70] Grassi L , Zachariae R, Caruso R, et al; ESMO Guidelines Committee. Insomnia in adult patients with cancer: ESMO clinical practice guideline. ESMO Open. 2023;8:102047. 38158225 10.1016/j.esmoop.2023.102047 PMC10774975

[71] Pinucci I , Maraone A, Tarsitani L, Pasquini M. Insomnia among cancer patients in the real world: optimising treatments and tailored therapies. Int J Environ Res Public Health. 2023;20:3785. 36900794 10.3390/ijerph20053785 PMC10001409

[72] Diaz-Frutos D , Baca-Garcia E, García-Foncillas J, López-Castroman J. Predictors of psychological distress in advanced cancer patients under palliative treatments. Eur J Cancer. 2016;25:608-615. 10.1111/ecc.12521 27271213

[73] Stern AF. The hospital anxiety and depression scale. Occup Med. 2014;64:393-394. 10.1093/occmed/kqu024 25005549

[74] Fann JR , Ell K, Sharpe M. Integrating psychosocial care into cancer services. J Clin Oncol. 2012;30:1178-1186. 22412139 10.1200/JCO.2011.39.7398

[75] Cassidy R , Singh NS, Schiratti P-R, et al Mathematical modelling for health systems research: a systematic review of system dynamics and agent-based models. BMC Health Serv Res. 2019;19:845. 31739783 10.1186/s12913-019-4627-7 PMC6862817

[76] Rimmer B , Brown MC, Sotire T, et al Characteristics and components of self-management interventions for improving quality of life in cancer survivors: a systematic review. Cancers. 2023;16:14. 38201442 10.3390/cancers16010014 PMC10777971

[77] Goldberg JI , Schulman-Green D, Hernandez M, Nelson JE, Capezuti E. Self-management interventions for psychological distress in adult cancer patients: a systematic review. West J Nurs Res. 2019;41:1407-1422. 31007160 10.1177/0193945919845104

[78] Boyd L , Baker E, Reilly J. Impact of a progressive stepped care approach in an improving access to psychological therapies service: an observational study. PloS One. 2019;14:e0214715. 30964883 10.1371/journal.pone.0214715 PMC6456251

[79] Noyes K , Zapf AL, Depner RM, et al Problem-solving skills training in adult cancer survivors: bright IDEAS-AC pilot study. Cancer Treat Res Commun. 2022;31:100552. 35358820 10.1016/j.ctarc.2022.100552 PMC9106910

[80] Michelson D , Malik K, Parikh R, et al Effectiveness of a brief lay counsellor-delivered, problem-solving intervention for adolescent mental health problems in urban, low-income schools in India: a randomised controlled trial. Lancet Child Adol Health. 2020;4:571-582. 10.1016/S2352-4642(20)30173-5 PMC7386943 32585185

[81] Zhang A , Park S, Sullivan JE, Jing S. The effectiveness of problem-solving therapy for primary care patients’ depressive and/or anxiety disorders: a systematic review and meta-analysis. J Am Board Fam Med. 2018;31:139-150. 10.3122/jabfm.2018.01.170270 29330248

[82] Kalter J , Verdonck‐de Leeuw IM, Sweegers MG, et al Effects and moderators of psychosocial interventions on quality of life, and emotional and social function in patients with cancer: an individual patient data meta‐analysis of 22 RCTs. Psycho-oncology. 2018;27:1150-1161. 29361206 10.1002/pon.4648 PMC5947559

[83] Zhou ES , Recklitis CJ. Internet‐delivered insomnia intervention improves sleep and quality of life for adolescent and young adult cancer survivors. Pediatr Blood Cancer. 2020;67:e28506. 10.1002/pbc.28506 32568460

[84] Clark PG. Decreasing psychological distress in cancer inpatients using FLEX care^®^: a pilot study. Soc Work Health Care. 2010;49:872-890. 20938880 10.1080/00981389.2010.499826

[85] Bower P , Gilbody S. Stepped care in psychological therapies: access, effectiveness and efficiency: narrative literature review. Br J Psychiatry. 2005;186:11-17. 15630118 10.1192/bjp.186.1.11

[86] Fitzgerald SP , Bean NG, Ruberu RP. A method of decision analysis quantifying the effects of age and comorbidities on the probability of deriving significant benefit from medical treatments. J Comorb. 2017;7:50-63. 29090189 10.15256/joc.2017.7.93 PMC5556438

[87] Clarke GM , Conti S, Wolters AT, Steventon A. Evaluating the impact of healthcare interventions using routine data. BMJ 2019;365:l2239. 31221675 10.1136/bmj.l2239 PMC6584784

[88] National Institute for Health and Care Excellence. Depression in adults: treatment and management: NICE guideline. National Institute for Health and Care Excellence. Updated June 29, 2022. Accessed February 12, 2024. https://www.nice.org.uk/guidance/ng222/resources/depression-in-adults-treatment-and-management-pdf-66143832307909

