# Supplementary material for: Stepped-care models for cancer symptom management: a systematic review of efficacy and cost-effectiveness
Source: J Natl Cancer Inst. 2025 Jun 25;118(1):26–48. doi: 10.1093/jnci/djaf153 (PMC12794235; doi:10.1093/jnci/djaf153)
Supplement: djaf153_Supplementary_Data [file djaf153_supplementary_data.zip › Supplementary File_Search Strategy.pdf]

Stepped-Care Models for Cancer Symptom Management: A Systematic Review of Efficacy and Cost-Effectiveness

Tasnim Abdalla[1], MPH, Gursharan K. Singh[2,3], BMedSc (Hons), PhD, Shiva Pouraliroudbaneh[4,5], MSc, Dorcas Serwaa[5], MSc, Michelle Peate[5], PhD

[1]Faculty of Health and Medical Science, The University of Western Australia, Western Australia, Australia
[2]Centre for Healthcare Transformation, Faculty of Health, Queensland University of Technology, Queensland, Australia
[3]Cancer and Palliative Care Outcomes Centre, School of Nursing, Queensland University of Technology, Queensland, Australia
[4]Centre for Adolescent Health- Murdoch Children's Research Institute- Royal Children Hospital, The University of Melbourne, Melbourne, Australia
[5]Department of Obstetrics, Gynaecology, and Newborn Health, Royal Women Hospital, The University of Melbourne, Melbourne, Australia

Correspondence to

Tasnim Abdalla, Faculty of Health and Medical Sciences, University of Western Australia

Perth, WA, Australia; Email: tasnim.abdalla@research.uwa.edu.au

Supplementary Material. Search Strategy

MEDLINE via Ovid	
Search#	Search terms
1	(exp neoplasms/) not (exp neoplasms, experimental or exp pregnancy complications, neoplastic/ or exp hamartoma)
2	(cancer* or leuk?emia or tumo?r* or neuroblastoma* or retinoblastoma* or neoplas* or malignan* or benign or lymphoma or sarcoma or rhabdomyosarcoma).ti,ab
3	1 or 2
4	("stepped care" or stepped-care or stepped or "matched care" or "adjunctive treatment" or "treatment tiering" or "adaptive treatment" or "sequential treatment" or "stratified care" or stage-based).ti,ab.
5	*patient care planning/ or exp progressive patient care/ or *patient care management/ or "delivery of health care"/ or exp disease management/ or *Needs Assessment/ or *Combined Modality Therapy/
6	4 or 5
7	3 and 6<br>*(english language and full text and humans and yr="2010 -Current" and (government publication or journal article or observational study or randomized controlled trial) and medline)*
**Cost-effectiveness Evidence**	
Search#	Search terms
8	*"Costs and Cost Analysis"/cl, ec, sn, td [Classification, Economics, Statistics & Numerical Data, Trends]
9	(cost-benefit or "cost benefit" or cost-consequence or "cost consequence" or "cost util*" or cost-util* or "cost eff*" or cost-eff* or cost* or econom* or "cost saving*" or "economic evaluation" or "cost evaluation" or "cost impact" or "economic impact" or "cost comparison" or "decision model*" or "economic model*").ti,ab.
10	8 or 9
11	limit 10 to<br>*(english language and full text and humans and yr="2010 -Current" and (government publication or journal article or observational study or randomized controlled trial))*
12	7 and 11
**APA PsycINFO via Ovid**	
Search#	Search terms
1	exp Neoplasms/
2	(cancer* or leuk?emia or tumo?r* or neuroblastoma* or retinoblastoma* or neoplas* or malignan* or benign or lymphoma or sarcoma or rhabdomyosarcoma).ti,ab
3	1 or 2
4	exp needs assessment/ or *patient centered care/ or exp treatment planning/ or *health care delivery/ or *disease management/

5	(stepped care or stepped-care or stepped or matched care or adjunctive treatment or treatment tiering or adaptive treatment or sequential treatment or stratified care or stage-based).ti,ab.
6	(stepped care or stepped-care or stepped or matched care or adjunctive treatment or treatment tiering or adaptive treatment or sequential treatment or stratified care or stage-based).mp.
7	4 or 5 or 6
8	3 and 7<br>*(full text and human and english language and journal article and yr="2010 - Current")*
**Cost-effectiveness Evidence**	
Search#	Search terms
9	"costs and cost analysis"/
10	(cost-benefit or cost benefit or cost-consequence or cost consequence or cost util* or cost-util* or cost eff* or cost-eff* or cost* or econom* or cost saving* or economic evaluation or cost evaluation or cost impact or economic impact or cost comparison or decision model* or economic model*). ti,ab
11	9 or 10
12	3 or 7 or 11<br>*(full text and human and english language and journal article and yr="2010 - Current")*

**EMBASE via Ovid**	
Search#	Search terms
1	exp neoplasm/
2	(cancer* or leuk?emia or tumo?r* or neuroblastoma* or retinoblastoma* or neoplas* or malignan* or benign or lymphoma or sarcoma or rhabdomyosarcoma).ti,ab
3	1 or 2
4	("stepped care" or stepped-care or stepped or "matched care" or "adjunctive treatment" or "treatment tiering" or "adaptive treatment" or "sequential treatment" or "stratified care" or stage-based).ti,ab.
5	exp patient centered care/ or *health care delivery/ or exp needs assessment/ or *patient care planning/ or *disease management/ or *treatment planning/
6	4 or 5
7	3 and 6<br>*limited (full text and human and english language and embase and yr="2010 -Current" and (article or article in press) and (journal or report))*
**Cost-effectiveness Evidence**	
Search#	Search terms
8	*"cost benefit analysis"/
9	(cost-benefit or "cost benefit" or cost-consequence or "cost consequence" or "cost util*" or cost-util* or "cost eff*" or cost-eff* or cost* or econom* or "cost saving*" or "economic evaluation" or "cost evaluation" or "cost impact" or "economic impact" or "cost comparison" or "decision model*" or "economic model*").ti,ab.
10	8 or 9

11	7 and 10<br>*limit to c(full text and human and english language and embase and yr="2010 - Current" and (article or article in press) and journal)*

**Web of Science via Institute for Scientific Information**	
Search#	Search terms
1	TOPIC: (cancer* or leuk?emia or tumo?r* or neuroblastoma* or retinoblastoma* or neoplas* or malignan* or benign or lymphoma or sarcoma or rhabdomyosarcoma)
2	TOPIC: (("stepped care" or stepped-care or stepped or "matched care" or "adjunctive treatment" or "treatment tiering" or "adaptive treatment" or "sequential treatment" or "stratified care" or stage-based)
3	1 and 2<br>*#1 AND #2 and 2023 or 2010 or 2011 or 2012 or 2013 or 2014 or 2015 or 2016 or 2017 or 2018 or 2019 or 2020 or 2021 or 2022 (Publication Years) and Article (Document Types) and English (Languages) and Article (Document Types) and Physiology or Psychology Multidisciplinary or Psychology or Psychiatry or Primary Health Care or Psychology Clinical or Psychology Applied (Web of Science Categories)*
4	TS=((cost-benefit or "cost benefit" or cost-consequence or "cost consequence" or "cost util*" or cost-util* or "cost eff*" or cost-eff* or cost* or econom* or "cost saving*" or "economic evaluation" or "cost evaluation" or "cost impact" or "economic impact" or "cost comparison" or "decision model*" or "economic model*”))<br><br>*and 2023 or 2022 or 2021 or 2020 or 2018 or 2019 or 2017 or 2015 or 2016 or 2014 or 2013 or 2011 or 2010 or 2012 (Publication Years) and Article or Data Paper or Early Access (Document Types) and English (Languages)*
5	3 and 4

**Cochrane Library**	
Search#	Search terms
1	MeSH descriptor: [Neoplasms] this term only
2	(cancer* or leuk*emia or tumo*r or neuroblastoma or retinoblastoma or malignant or neoplasm* or malignancies or malignant neoplasm or benign neoplasms or neoplasia or lymphoma or sarcoma or rhabdomyosarcoma):ti
3	(cancer* or leuk*emia or tumo*r or neuroblastoma or retinoblastoma or malignant or neoplasm* or malignancies or malignant neoplasm or benign neoplasms or neoplasia or lymphoma or sarcoma or rhabdomyosarcoma):ab
4	1 or 2 or 3
5	MeSH descriptor: [Needs Assessment] 1 tree(s) exploded<br>MeSH descriptor: [Patient-Centered Care] this term only<br>MeSH descriptor: [Combined Modality Therapy] this term only<br>MeSH descriptor: [Patient Care Planning] this term only<br>MeSH descriptor: [Progressive Patient Care] this term only<br>MeSH descriptor: [Disease Management] this term only<br>MeSH descriptor: [Delivery of Health Care] this term only<br>#1 or #2 or #3 or #4 or #5 or #6 or #7

6	("stepped care" or "stepped-care" or stepped or "matched care" or "adjunctive treatment*" or "treatment tiering" or "adaptive treatment*" or "sequential treatment*" or "stratified care" or "stage-based"):ti
7	("stepped care" or "stepped-care" or stepped or "matched care" or "adjunctive treatment*" or "treatment tiering" or "adaptive treatment*" or "sequential treatment*" or "stratified care" or "stage-based"):ab
8	5 or 6 or 7
7	4 and 8<br>*with Cochrane Library publication date from Jan 2010 to Dec 2022, in Trials*
**Cost-effectiveness Evidence**	
Search#	Search terms
14	MeSH descriptor: [Costs and Cost Analysis] explode all trees
15	(cost-benefit or "cost benefit" or cost-consequence or "cost consequence" or "cost util*" or cost-util* or "cost eff*" or cost-eff* or cost* or econom* or "cost saving*" or "economic evaluation" or "cost evaluation" or "cost impact" or "economic impact" or "cost comparison" or "decision model*" or "economic model*"):ti
16	(cost-benefit or "cost benefit" or cost-consequence or "cost consequence" or "cost util*" or cost-util* or "cost eff*" or cost-eff* or cost* or econom* or "cost saving*" or "economic evaluation" or "cost evaluation" or "cost impact" or "economic impact" or "cost comparison" or "decision model*" or "economic model*"):ab
17	14 or 15 or 16<br>*with Cochrane Library publication date from Jan 2010 to Dec 2021*
18	13 and 17<br>*with Cochrane Library publication date from Jan 2010 to Dec 2022, in Trials*
**National Health Service Economic Evaluation Database**	
Search#	Search terms
1	(cancer* or leuk*emia or tumo*r or neuroblastoma or retinoblastoma or malignant or neoplasm* or malignancies or malignant neoplasm or benign neoplasms or neoplasia or lymphoma or sarcoma or rhabdomyosarcoma) AND (stepped care or stepped-care or stepped or matched care or adjunctive treatment* or treatment tiering or adaptive treatment* or sequential treatment* or stratified care or stage-based)
2	(cancer* or leuk*emia or tumo*r or neuroblastoma or retinoblastoma or malignant or neoplasm* or malignancies or malignant neoplasm or benign neoplasms or neoplasia or lymphoma or sarcoma or rhabdomyosarcoma) AND (stepped care or stepped-care or stepped or matched care or adjunctive treatment* or treatment tiering or adaptive treatment* or sequential treatment* or stratified care or stage-based) AND (cost-benefit or cost benefit or cost-consequence or cost consequence or cost util* or cost-util* or cost eff* or cost-eff* or cost* or econom* or cost saving* or economic evaluation or cost evaluation or cost impact or economic impact or cost comparison or decision model* or economic model*)
**EconLit via EBSCO**	
Search#	Search terms

1	TX (cancer* or leuk*emia or tumo*r or neuroblastoma or retinoblastoma or malignant or neoplasm* or malignancies or malignant neoplasm or benign neoplasms or neoplasia or lymphoma or sarcoma or rhabdomyosarcoma) AND TX (stepped care or stepped-care or stepped or matched care or adjunctive treatment* or treatment tiering or adaptive treatment* or sequential treatment* or stratified care or stage-based)
